# Targeting the HuR/E2F7 axis synergizes with bortezomib against multiple myeloma

**DOI:** 10.1038/s41401-025-01529-3

**Published:** 2025-03-25

**Authors:** Ming-yuan Jia, Chao Wu, Ze Fu, Wen-bin Xu, Jia Liu, Cheng-yu Wu, Xin-yi Zeng, Ying-li Wu, Hua Yan

**Affiliations:** 1 Shanghai Institute of Hematology, State Key Laboratory of Medical Genomics, National Research Center for Translational Medicine at Shanghai, Ruijin Hospital, Shanghai Jiao Tong University School of Medicine, Shanghai, 200025 China; 2 Department of General Practice, Ruijin Hospital, Shanghai Jiao Tong University School of Medicine, Shanghai, 200025 China; 3 Department of Hematology, Huadong Hospital Affiliated with Fudan University, Shanghai, 200040 China; 4 Hongqiao International Institute of Medicine, Shanghai Tongren Hospital/Faculty of Basic Medicine, Chemical Biology Division of Shanghai Universities E-Institutes, Key Laboratory of Cell Differentiation and Apoptosis of the Chinese Ministry of Education, Shanghai Jiao Tong University School of Medicine, Shanghai, 200336 China

**Keywords:** multiple myeloma, HuR, E2F7, CMLD-2, bortezomib

## Abstract

Multiple myeloma (MM) is a malignant hematological disease caused by the proliferation of abnormal plasma cells in the bone marrow and is still incurable. Relapse and drug resistance are common in MM. New therapeutic targets are urgently needed for MM treatment. Human antigen R (HuR) has been reported to play an important role in the malignant biological behavior of a variety of tumors, but its role in MM remains unclear. In this study, we found that HuR was highly expressed in MM patients and associated with a poor prognosis by analyzing public datasets. We found that targeting HuR with short hairpin RNA (shRNA) or its inhibitor CMLD-2 had significant anti-MM effects both in vitro and in vivo. The overexpression of HuR promotes MM cell proliferation in vitro and in vivo. Moreover, we demonstrated that bortezomib drug sensitivity increased and decreased with the knockdown and overexpression of HuR, respectively. This result provides a rationale for our subsequent combination of CMLD-2 with bortezomib in the treatment of MM. To further explore the mechanism of HuR in MM, we performed RNA sequencing and identified its downstream molecule, E2F7. HuR upregulated E2F7 expression by increasing the stability of its mRNA in MM cells. Higher levels of E2F7 were associated with a poorer prognosis. E2F7 knockdown had anti-MM effects in vitro and in vivo. E2F7 overexpression partially rescued the cell proliferation inhibition and apoptosis caused by targeting HuR in MM cells. We subsequently demonstrated that CMLD-2 synergized with the anti-MM effect of bortezomib both in vitro and in vivo. In conclusion, targeting the HuR/E2F7 axis synergizes with bortezomib against MM. Therefore, the HuR/E2F7 axis may serve as a promising therapeutic target for MM.

## Introduction

Multiple myeloma (MM) is a malignant proliferative disorder of plasma cells, accounting for approximately 10% of all hematologic malignancies [1, 2]. Current therapies for MM mainly include proteasome inhibitors (bortezomib, carfilzomib, and ixazomib), immunomodulators (lenalidomide and pomalidomide), B-cell maturation antigen (BCMA)- or CD38-targeted therapies, and chimeric antigen receptor T (CAR-T) cell therapies [3–6]. Although these therapies have significantly improved the prognosis of MM patients, disease relapse and drug resistance continue to occur in MM patients. Thus, new targets are needed for the treatment of MM.

As important molecules of gene posttranscriptional modification, RNA-binding proteins (RBPs) play important roles in cancer by affecting mRNA modification, splicing, translation and stability [7]. Multiple studies have reported that human antigen R (HuR), an RBP, plays an important role in a variety of tumors [8–14]. HuR plays a role in other tumors mainly by stabilizing or modifying target gene mRNAs, thereby affecting tumor cell proliferation, metastasis, energy metabolism, drug resistance, and immune escape [15–21]. The major targeted mRNAs of HuR contain AU-rich elements in their 3′-untranslated region (3′-UTR) [22–24]. However, the expression and biological functions of HuR in MM remain unclear.

E2F7 is an atypical member of the E2F transcription factor family [25]. It has been reported that E2F7 can competitively bind to the corresponding target gene with E2F1, thus playing a transcriptional inhibitory role [26]. E2F7 is highly expressed in a variety of tumors and is associated with a poor prognosis by influencing tumor cell proliferation, apoptosis, the cell cycle, and drug sensitivity [27–30]. Currently, the role of E2F7 in cancer is still controversial. Specifically, the clinical significance of E2F7 and its role in MM need further investigation.

In the present study, we observed increased levels of HuR in MM patients, and higher levels of HuR or E2F7 were correlated with poor overall survival. Moreover, the knockdown of HuR or E2F7 had significant anti-MM effects in vitro and in vivo. Importantly, HuR knockdown increased the sensitivity of MM cells to bortezomib, and CMLD-2 synergized with the anti-MM effect of bortezomib both in vitro and in vivo. In addition, HuR can bind to the E2F7 3′-UTR and upregulate E2F7 expression by increasing its mRNA stability. Therefore, our results indicate that the HuR/E2F7 axis plays a key role in the malignancy of MM and provides a potential therapeutic target for MM.

## Materials and methods

### Cell culture and reagents

The human MM cell lines NCI-H929, U266, MM.1S, RPMI 8226, and HEK293T were purchased from the American Type Culture Collection (Manassas, VA, USA). LP-1 and OPM2 were obtained from the German Collection of Microorganisms and Cell Cultures (Braunschweig, Germany). These cell lines were routinely tested for mycoplasma contamination and were negative. All cells were cultured at 37 °C in a humidified chamber with 5% CO_2_. MM cells were suspended in RPMI-1640 medium, and HEK293T cells were suspended in DMEM supplemented with 10% fetal bovine serum (Sigma Aldrich, St. Louis, MO), 100 μg/ml streptomycin (YEASEN, China), and 100 IU/ml penicillin (Invitrogen).

CMLD-2, bortezomib, lenalidomide, dexamethasone, and actinomycin D were purchased from MCE (MedChemExpress, USA), dissolved in dimethyl sulfoxide (Sigma, St. Louis, MO), and stored in the dark at −20 °C.

### Patient samples

Density gradient centrifugation with Ficoll-Paque Plus (Sigma‒Aldrich, St. Louis, MO) was used to isolate BMMCs from MM patients and PBMCs from healthy donors. CD138-positive plasma cells from BMMCs were subsequently isolated with anti-CD138 microbeads (Miltenyi Biotec, Bergisch Gladbach, Germany) according to the manufacturer’s instructions.

### Bioinformatics

The gene expression and survival data of MM patients were obtained from the Multiple Myeloma Research Foundation (MMRF) CoMMpass study (https://xena.ucsc.edu). The gene expression data of GSE6477, GSE57317, GSE46816, and GSE9782 and the survival data of GSE4581 and GSE2658 were obtained from the Gene Expression Omnibus (GEO, http://www.ncbi.nlm.nih.gov/geo/). The MSIGDB database was used for gene enrichment analysis (http://software.broadinstitute.org/gsea/msigdb). An |NES| > 1 and a *P* value < 0.05 were considered to indicate significant enrichment.

### Cell proliferation assay

Cell proliferation was detected via a Cell Counting Kit-8 (CCK-8) assay (New Cell and Molecular Biotech, Suzhou, China). MM cells were seeded in triplicate in 96-well plates. The absorbance at 450 nm was measured using a microplate reader (BioTek Instruments, Winooski, VT) after the addition of the CCK-8 reagent. The combined effect was assessed via SynergyFinder (https://synergyfinder.fimm.fi/) [31]; a ZIP synergy score > 10, between −10 and 10, and < −10 indicated synergism, an additive effect, and antagonism, respectively.

### Detection of the cell cycle

MM cells were harvested, washed, fixed overnight at −20 °C with 70% ethanol, and incubated in the dark with PI/RNase staining buffer (BD Biosciences). The fluorescence of the stained cells was measured using a CytoFLEX cytometer (Beckman, Brea, CA). The cell cycle distribution was determined by a PI staining assay. The distribution of each cell cycle phase was analyzed by ModFit software.

### Detection of cell apoptosis

Detection of cell apoptosis was performed by flow cytometry via an Annexin V/PI double-staining kit (BD Biosciences, San Diego, CA, USA). Early apoptotic cells (Annexin V positive and PI negative) and late apoptotic cells (Annexin V positive and PI positive) were classified as apoptotic cells according to the manufacturer’s instructions. The results were analyzed with FlowJo 10.0 software.

### Western blotting

Appropriate amounts of cells were removed, washed, and lysed. Then, equal amounts of protein extracts were subjected to electrophoresis on sodium dodecyl sulfate polyacrylamide gels and transferred to nitrocellulose membranes. Following blocking with 5% skim milk, the membranes were incubated overnight at 4 °C with primary antibodies and then incubated with horseradish peroxidase (HRP)-conjugated secondary antibodies. The signals were visualized using a chemiluminescence phototope-HRP kit (Cell Signaling Technology, Danvers, MA). Antibodies against PARP1 (catalog, 13371-1-AP), caspase 3 (catalog, 19677-1-AP), cyclin D1 (catalog, 60186-1-Ig), CDK4 (catalog, 66950-1-Ig), β-actin (catalog, 66009-1-Ig), HuR (catalog, 66549-1-Ig), and E2F7 (catalog, 24489-1-AP) were used.

### Quantitative reverse transcription real-time PCR (qRT‒PCR)

Total cellular RNA was extracted via TRIzol reagent (Invitrogen, Carlsbad, CA). After quantification, the RNA was converted to cDNA via an Evo M-MLV RT Kit with a gDNA Clean for qPCR Kit (Accurate Biology, Changsha, China). cDNA was used as a template for qRT‒PCR via a SYBR Green Premix Pro Taq HS qPCR Kit (Accurate Biology, Changsha, China). The expression levels of all the genes were normalized to that of β-actin and calculated using the 2^−ΔΔCt^ method. The qRT‒PCR primers for the E2F family and HuR are listed in Supplementary Table 1.

### RNA-binding protein immunoprecipitation (RIP) experiments

Appropriate amounts of NCI-H929 and OPM2 cells were used for RIP experiments with an anti-HuR antibody, IgG, and Merck Millipore Magna RIP kit (17–700, Merck Millipore) according to the manufacturer’s instructions. The quantification of the RNA obtained from the RIP experiments was conducted using a Nanodrop 2000 (Thermo Fisher). Then, qRT‒PCR was performed using the following primers. E2F7-F: 5'-AAAGGGACTATTCCGACCCAT-3'; E2F7-R: 5'-ACTTGGATAGCGAGCTAGAAACT-3'; β-actin-F: 5'-CATGTACGTTGCTATCCAGGC-3'; and β-actin-R: 5'-CTCCTTAATGTCACGCACGAT-3'.

### Plasmid construction and lentiviral packaging

The sequences of shRNAs against HuR or E2F7 were subsequently cloned and inserted into the lentiviral pLKO.1-puro vector. A FLAG tag was added to the C-terminus of the E2F7 coding sequence (CDS). The overexpression plasmids (E2F1-OE, E2F2-OE, E2F3-OE, E2F8-OE, E2F7-OE, and HuR-OE) were constructed by inserting CDS fragments (E2F1, E2F2, E2F3, E2F8, E2F7-flag or HuR) obtained by PCR into the pLVX-puro vector. The above-constructed plasmids were cotransfected into HEK293T cells with the packaging plasmids psPAX2 and pMD2G. Infectious lentiviruses were harvested and filtered through 0.45-μm PVDF filters. The shRNA sequences of HuR were as follows: shHuR#1: 5′-CGTGGATCAGACTACAGGTTT-3′ and shHuR#2: 5′-GCAGCATTGGTGAAGTTGAAT-3′. The shRNA sequences of E2F7 were as follows: shE2F7#1: 5′-GCAGTCTCCTGCAGGATTAAA-3′ and shE2F7#2: 5′-GTGCTGCCAGCCCAGATATAA-3′.

### RNA decay assay

NCI-H929 and OPM2 cells transfected with shNC or shHuR lentiviruses were treated with actinomycin D (5 μg/mL) (MCE, HY-17559) for 0, 2, 4, or 6 h. Total mRNA was subsequently extracted, and qRT‒PCR was performed to quantify relative mRNA levels (relative to 0 h).

### Luciferase reporter assay

PCR was used to amplify the 3′-UTR of human E2F7, which contains the putative HuR binding site. A QuickMutation™ Site-Directed Mutagenesis Kit (Beyotime, Shanghai, China) was used to create the mutant 3′-UTR fragment of E2F7. The 3′-UTR wt and 3′-UTR mut fragments of E2F7 were then cloned and inserted into the Promega psiCHECK2 luciferase vector. HEK293T cells were cotransfected with the luciferase reporter plasmids and the plvx-HuR or plvx-NC plasmid. A Dual Luciferase Assay Kit (YEASEN, China) was used to detect luciferase activity at 48 h after cotransfection. The relative luciferase activity was normalized to firefly luciferin activity.

### RNA sequencing (RNA-seq)

After NCI-H929 cells were transfected with shNC or shHuR for 48 h, total RNA was extracted from the cells as described above using TRIzol Reagent (Invitrogen, USA). The RNA sequences obtained from the above RNA samples were subsequently sent to Personal Biotechnology Co., Ltd (Shanghai, China) on an Illumina NovaSeq platform.

### Animal experiments

Four-week-old NOG mice were purchased from Vital River Laboratory Animal Technology Co., Ltd (Beijing, China). Following the infection of NCI-H929 cells or OPM2 cells with lentiviruses (shNC, shHuR, vector, or HuR-OE) for 48 h, 3 × 10^6^ cells were resuspended in PBS and Corning Matrigel as described above and then subcutaneously injected into the right flanks of NOG mice. At the end of the experiment, the mice were sacrificed, and the tumor tissues were weighed.

After collection, 3 × 10^6^ NCI-H929 cells were mixed with Matrigel and subcutaneously injected into the right flank of each mouse. When the tumors were measurable, the mice were randomly divided into the CMLD-2 treatment group, bortezomib treatment group, combination group, and control group (5 mice in each group). In the treatment groups, the mice received 20 mg/kg CMLD-2 via intraperitoneal injections every other day for 14 days or intravenous injections of 1 mg/kg bortezomib on days 1, 4, 8, and 11. Tumor size and mouse weights were measured every 2 days using a caliper. The tumor volume (TV) was calculated using the formula TV = Length × Width^2^/2. At the end of the experiment, the mice were sacrificed by carbon dioxide asphyxiation, and the tumor tissues were weighed, measured, photographed, and then fixed with 4% formalin solution or stored at −80 °C for subsequent experiments.

### Statistical analysis

The data are shown as the means ± standard deviations (SDs). Two-tailed paired or unpaired Student’s *t*-tests were used to compare differences between two groups. One-way analysis of variance (ANOVA) was used to compare differences among three or more groups. The overall survival of patients was evaluated using the Kaplan‒Meier method, and significant differences in survival curves were compared using the log-rank test. Statistical analysis was performed using GraphPad Prism 9.0 software. Statistical differences are presented as *P < 0.05, **P < 0.01, ***P < 0.001, and ****P < 0.0001.

## Results

### HuR is highly expressed and associated with a poor prognosis in MM

**Fig. 1:**
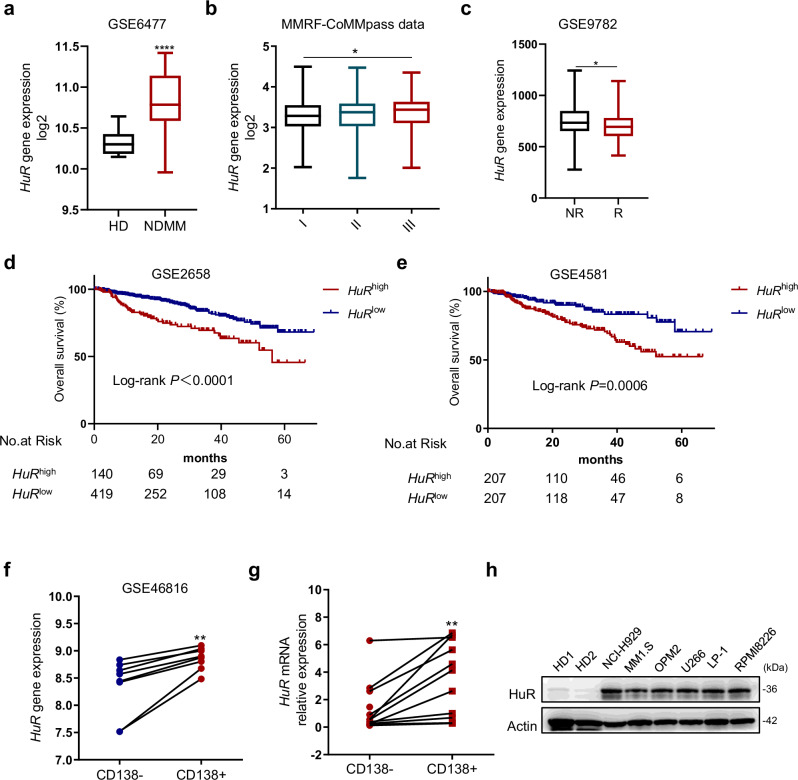
HuR is highly expressed and associated with a poor prognosis in MM. **a** The HuR expression data from the GSE6477 dataset were compared among CD138-positive bone marrow plasma cells from healthy donors (HDs, *n* = 15) and newly diagnosed multiple myeloma (NDMM) patients (*n* = 69). **b** In the MMRF-CoMMpass database, HuR expression in CD138-positive BMMCs was analyzed in patients with ISS stage I (*n* = 281), ISS stage II (*n* = 298), and ISS stage III (*n* = 258) disease. **c** Comparison of HuR expression between the NR (bortezomib nonresponders, *n* = 84) and R (bortezomib responders, *n* = 85) groups in the GSE9782 dataset. **d**, **e** In the GSE2658 and GSE4581 datasets, the log-rank test was used to statistically analyze overall survival in MM patients with high HuR expression (HuR^high^) and low HuR expression (HuR^low^). **f** The eight paired sets of CD138^+^ and CD138^−^ cells were analyzed for HuR expression in the GSE46816 database. **g** The mRNA expression levels of HuR were measured by qRT‒PCR in CD138^+^ and CD138^−^ cell populations obtained from the BMMCs of MM patients. **h** Protein levels of HuR in MM cell lines (NCI-H929, MM.1S, OPM2, U266, LP-1 and RPMI 8266) and two healthy human PBMCs (HD1 and HD2) detected by Western blotting (WB). The data are shown as the means ± standard deviations (SDs), and statistical analysis was performed via Student’s *t*-test (**a**, **c**, **f**, **g**) or one-way analysis of variance (ANOVA) (**b**). *P < 0.05, **P < 0.01, ****P < 0.0001.

To investigate the expression of HuR in MM and its correlation with MM patient prognosis, we analyzed the expression of HuR in healthy donors (HDs) and newly diagnosed MM (NDMM) patients in the GSE6477 database and found that HuR expression levels were higher in NDMM patients than in HDs (Fig. 1a). According to the Multiple Myeloma Research Foundation (MMRF) CoMMpass database, the HuR expression level was higher in MM patients with International Staging System (ISS) stage III disease than in those with ISS stage I disease (Fig. 1b). Moreover, the GSE9872 data revealed that MM patients who responded better to bortezomib had lower HuR levels (Fig. 1c). To further identify the associations between HuR and clinical outcomes in MM patients, data from the GSE2658 and GSE4581 datasets were analyzed, and higher HuR mRNA levels were associated with poorer overall survival (Fig. 1d, e). In eight pairs of myeloma cell lines from the GSE46816 dataset, the HuR expression level was greater in CD138-positive cells than in CD138-negative cells (Fig. 1f). To confirm these findings, we evaluated the mRNA levels of HuR in CD138-positive and CD138-negative paired samples from MM bone marrow mononuclear cells (BMMCs) via qRT‒PCR and analyzed the protein levels of HuR in PBMCs from healthy donors and six different MM cell lines by Western blotting (WB). Consistently, CD138-positive cells presented greater expression of HuR than did CD138-negative cells. Compared with that in cells from healthy donors, HuR was highly expressed in MM cell lines (Fig. 1g, h). These results demonstrate that the upregulation of HuR in MM is correlated with a poor prognosis and sensitivity to bortezomib.

### HuR knockdown suppresses proliferation, facilitates apoptosis, and induces G_0_/G_1_ phase arrest in MM cells

**Fig. 2:**
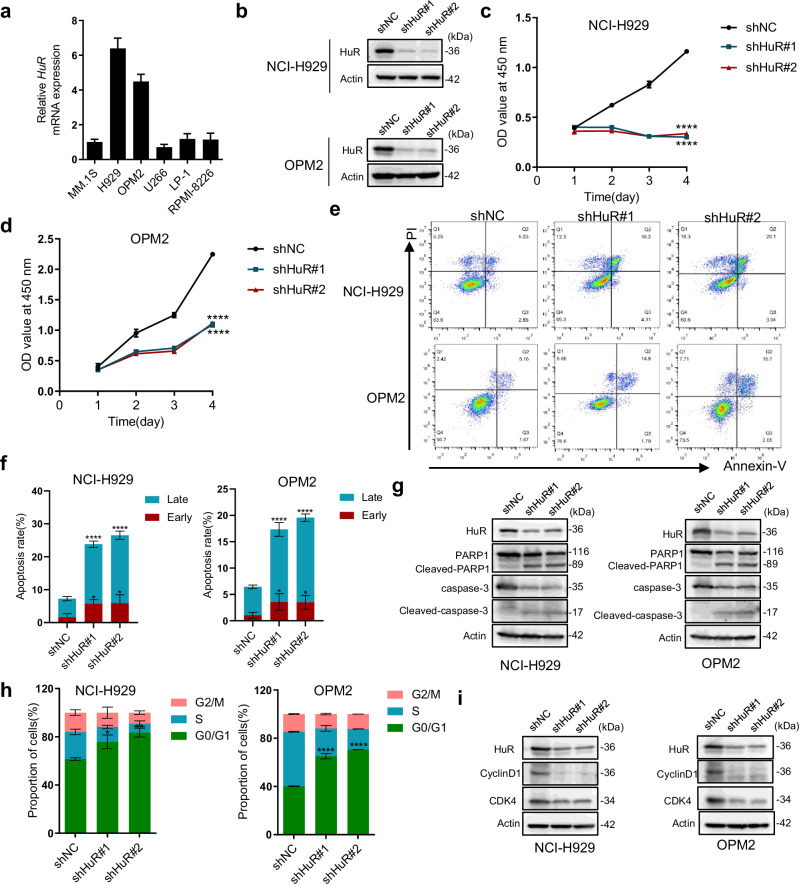
HuR knockdown suppresses proliferation, facilitates apoptosis, and induces G_o_/G_1_ phase arrest in MM cells. **a** Relative expression of HuR measured by qRT‒PCR in six MM cell lines. **b** The knockdown efficiency of HuR in NCI-929 and OPM2 cells was detected by WB. **c**, **d** CCK-8 assays were used to detect the proliferation of NCI-H929 and OPM2 cells after the knockdown of HuR for 4 consecutive days. **e** After 48 h of lentiviral infection for HuR knockdown, the apoptosis of NCI-H929 and OPM2 cells was detected by flow cytometry using Annexin V/PI double staining. **f** Early apoptotic cells (Annexin V positive and PI negative) and late apoptotic cells (Annexin V positive and PI positive) were used to analyze the apoptosis rate. **g** Apoptosis-related protein (PARP1, cleaved PARP1, caspase 3, and cleaved caspase 3) levels following HuR knockdown in NCI-H929 and OPM2 cells were measured using WB. **h** In NCI-H929 and OPM2 cells, flow cytometry was used to detect the cell cycle after HuR knockdown. **i** G_0_/G_1_ phase-associated protein (CDK4, cyclin D1) levels following HuR knockdown in NCI-H929 and OPM2 cells were determined using WB. The data are shown as the means ± SD and were analyzed by one-way ANOVA. ****P < 0.0001.

To further explore the biological function of HuR in MM, we first examined the relative expression abundance of HuR in six different MM cell lines (Fig. 2a). We performed subsequent loss-of-function experiments using NCI-H929 and OPM2 cells with relatively high HuR expression levels. Under the premise of clarifying the efficiency of HuR knockdown, we found that HuR knockdown in NCI-H929 and OPM2 cells significantly inhibited cell proliferation (Fig. 2b–d).

Following detection of apoptosis by flow cytometry, we found that HuR knockdown induced apoptosis in NCI-H929 and OPM2 cells, accompanied by increases in the levels of the apoptosis-related proteins cleaved PARP1 and cleaved caspase 3 compared with those in the shNC group (Fig. 2e–j). Given that HuR knockdown did not result in a particularly significant rate of apoptosis or significant inhibition of proliferation, we examined the effects of HuR knockdown on the MM cell cycle by flow cytometry and found that HuR knockdown resulted in significant G_0_/G_1_ arrest in NCI-H929 and OPM2 cells, accompanied by decreased protein levels of the G_0_/G_1_ phase-related proteins cyclin D1 and CDK4 (Figs. 2h, i and S1a). In summary, HuR knockdown had a significant anti-MM effect in vitro, indirectly indicating that HuR plays an important role in maintaining the malignant proliferation and survival of MM cells.

### HuR knockdown shows anti-MM effects in vivo

**Fig. 3:**
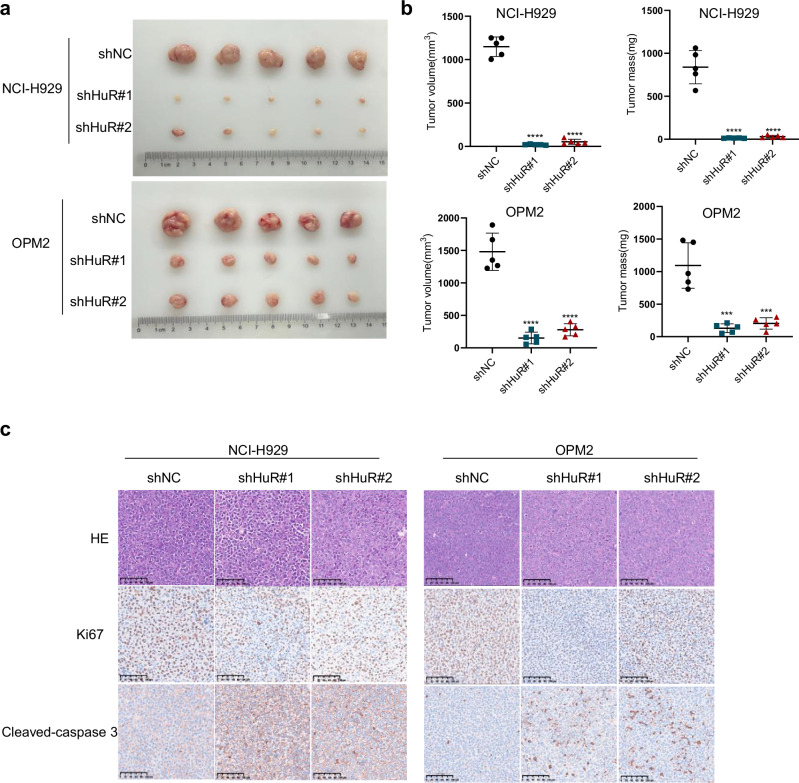
HuR knockdown shows anti-MM effects in vivo. **a** NCI-H929 and OPM2 cells transfected with lentiviruses (shNC, shHuR#1 or shHuR#2) were inoculated subcutaneously into NOG mice to construct xenograft mouse models. These subcutaneous tumors were dissected from each group of mice and photographed at the end of the experiment (*n* = 5). **b** Subcutaneous tumors from the above groups of mice were measured, weighed, and compared. **c** Subcutaneous tumors from three groups of mice, shNC, shHuR#1, and shHuR#2, were embedded in paraffin and stained with hematoxylin and eosin (HE), Ki67, and cleaved caspase 3 antibody (scale 100 μm). The data are shown as the means ± SD and were analyzed by one-way ANOVA. ***P < 0.001, ****P < 0.0001.

On the basis of the above studies, to further investigate the biological role of HuR in MM, we used NOG mice to further simulate the growth of MM in vivo. First, we subcutaneously inoculated the NCI-H929 and OPM2 cell lines transfected with lentiviruses (shNC, shHuR#1, or shHuR#2) into the flanks of NOG mice to construct xenograft mouse models (Fig. S2a). At the end of the experiment, we dissected subcutaneous tumors from the mice and then measured and weighed the tumors. The results revealed that the tumor volume and weight in the HuR knockdown group were significantly lower than those in the shNC group (Fig. 3a, b). Then, we lysed the tumors to clarify the knockdown efficiency of HuR (Fig. S2b). The results indicated that HuR knockdown significantly inhibited the proliferation of MM cells in vivo. We then subjected the tumors from each group to immunohistochemical (IHC) examination and found that the proportion of Ki67-positive cells in the tumors from the shHuR groups was significantly lower and that the proportion of cleaved caspase 3-positive cells was significantly greater than that in the shNC group (Fig. 3c). Taken together, these findings indicate that HuR knockdown significantly inhibits tumor proliferation, facilitates apoptosis, and has a significant anti-MM effect in vivo.

### CMLD-2, an inhibitor of HuR, has anti-MM effects in vitro

**Fig. 4:**
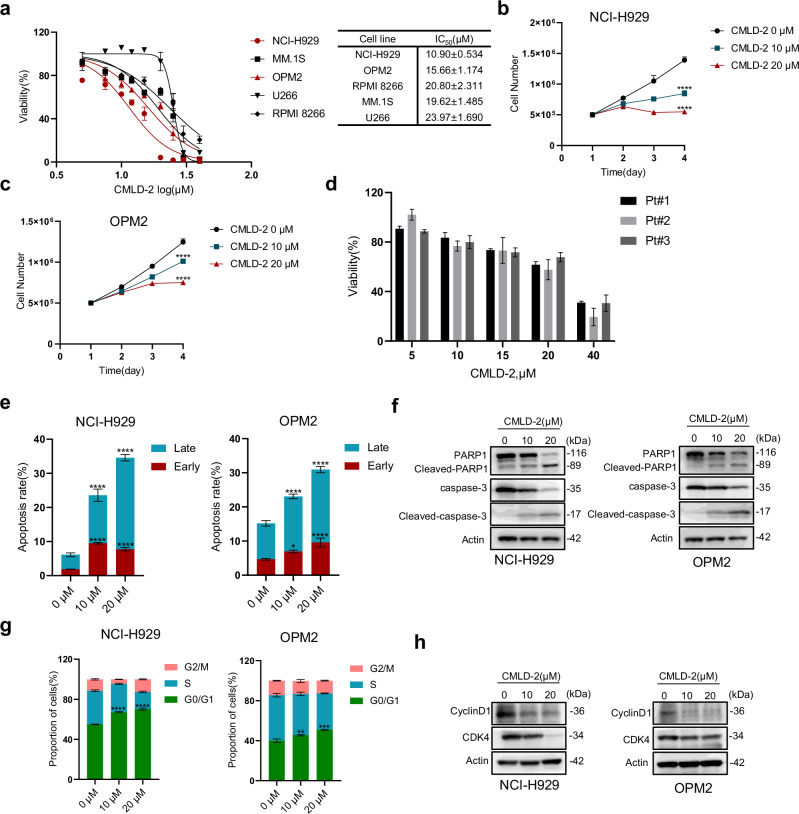
CMLD-2, an inhibitor of HuR, has anti-MM effects in vitro. **a** Six different MM cell lines were treated with CMLD-2 (0–40 μM) for 48 h, and the viability of these cells was tested by CCK-8 assays. IC_50_ values from triplicate independent experimental data were calculated from dose‒response curves and are presented as the means ± SDs. **b**, **c** CMLD-2 (0 μM, 10 μM, or 20 μM) was added to NCI-H929 and OPM2 cells, after which the cell numbers were recorded for 4 consecutive days after treatment, and the significant differences between the two groups on day 4 were analyzed. **d** BMMCs isolated from three MM patients (Pt #1, #2, and #3) were treated with CMLD-2 at the indicated concentrations for 48 h, and cell viability was then assessed via CCK-8 assays. **e** NCI-H929 and OPM2 cells were treated with CMLD-2 (0 μM, 10 μM, or 20 μM) for 48 h, and the early and late apoptosis rates were analyzed. **f** NCI-H929 and OPM2 cells were treated with CMLD-2 (0 μM, 10 μM, or 20 μM) for 48 h, and the levels of apoptosis-related proteins (PARP1, cleaved PARP1, caspase 3, and cleaved caspase 3) were measured by WB. **g**, **h** NCI-H929 and OPM2 cells were treated with CMLD-2 (0 μM, 10 μM, or 20 μM) for 48 h. The cell cycle distribution of NCI-H929 and OPM2 cells was measured by flow cytometry, and the levels of G0/G1 phase-related proteins (CDK4 and cyclin D1) were measured by WB. The data are shown as the means ± SD and were analyzed by one-way ANOVA. *P < 0.05, **P < 0.01, ***P < 0.001, ****P < 0.0001.

To further validate the translational feasibility of HuR for MM clinical treatment, we performed further experiments using a small molecule inhibitor of HuR, CMLD-2, which mainly affects the binding of HuR to target genes, thereby inhibiting HuR [16]. We tested the viability of six different MM cell lines treated with the indicated concentrations of CMLD-2 for 48 h. We found that CMLD-2 showed dose-dependent cytotoxicity against MM cell lines, with IC_50_ values ranging from 10.90 ± 0.534 μM to 23.97 ± 1.690 μM (Fig. 4a). Next, we selected NCI-H929 and OPM2 cells, which are relatively sensitive to CMLD-2, for subsequent experiments. Different concentrations of CMLD-2 were added to the above two cell lines, followed by recording of cell proliferation for 4 consecutive days, and the results showed that CMLD-2 significantly inhibited MM cell proliferation (Fig. 4b, c). Furthermore, CMLD-2 significantly inhibited the proliferation of BMMCs from MM patients but had little toxic effect on PBMCs from healthy donors (Figs. 4d and S3a). To further investigate the effects of CMLD-2 on MM cells, we next performed apoptosis and cell cycle-related studies in NCI-H929 and OPM2 cells 48 h after the addition of CMLD-2 at the indicated concentrations. We found that CMLD-2 induced apoptosis and caused cycle arrest in the G_0_/G_1_ phase in MM cells, accompanied by an increase in the levels of the apoptosis-related proteins cleaved PARP1 and caspase 3 and a decrease in the levels of the G_0_/G_1_ phase-related proteins CDK4 and cyclin D1 (Figs. 4e–h, and S3b, c). In summary, CMLD-2 has a significant anti-MM effect in vitro, providing a good experimental basis for subsequent in vivo experiments in MM mouse models and for the clinical treatment of MM.

### Overexpression of HuR promotes MM cell proliferation in vitro and in vivo

To further explore the function of HuR in MM, we performed gain-of-function experiments in NCI-H929 and OPM2 cells. Under the premise of clarifying the efficiency of HuR overexpression, we found that the overexpression of HuR significantly promoted MM cell proliferation (Fig. S4a–c). However, HuR overexpression had little effect on MM cell apoptosis (Fig. S4d). To further explore the effect of HuR on MM cell proliferation in vivo, we subcutaneously inoculated NCI-H929 and OPM2 cell lines transfected with lentiviruses (vector or HuR-OE) into the flanks of NOG mice to construct xenograft mouse models. The results revealed that the tumor volume and weight in the HuR overexpression group were significantly greater than those in the vector group (Fig. S4e, f). These data suggest that HuR promotes MM cell proliferation in vitro and in vivo.

### E2F7 is the main downstream molecule of HuR in MM cells

**Fig. 5:**
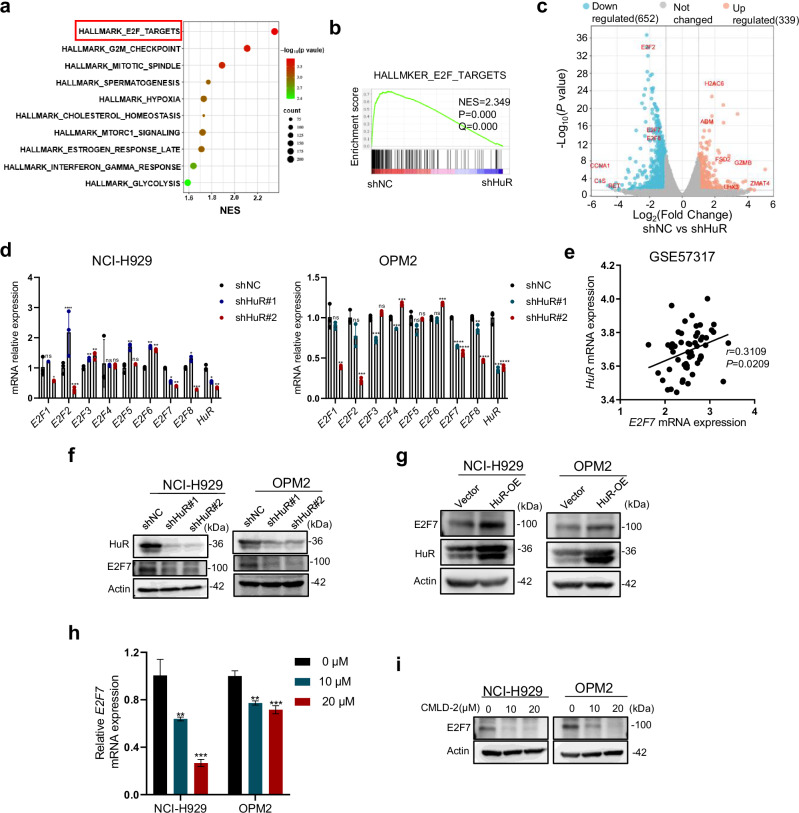
E2F7 is the main downstream molecule of HuR in MM cells. **a**, **b** RNA sequencing data from shHuR- versus shNC-treated NCI-H929 cells were analyzed, and gene set enrichment analysis (GSEA) of differentially expressed genes (DEGs) was performed using the online Molecular Signature Database (MSigDB). Bubble plots were generated for the top ten gene sets according to normalized enrichment scores (NESs). GSEA plots of E2F targets in the shNC and shHuR groups were also generated. **c** Volcano plot of the transcriptome DEGs in the NCI-H929 shNC and shHuR groups, including upregulated, downregulated, and nonsignificantly changed genes (*P* < 0.05, fold change > 1.5). **d** After knockdown of HuR in NCI-H929 and OPM2 cells, E2F1-E2F8 mRNA expression levels were measured via qRT‒PCR. **e** The association between HuR and E2F7 was analyzed using GSE57317 data from MM patients. **f**, **g** The protein levels of E2F7 were detected in NCI-H929 and OPM2 cells with HUR knockdown and overexpression, respectively. **h**, **i** NCI-H929 and OPM2 cells were treated with CMLD-2 (0 μM, 10 μM, or 20 μM) for 48 h, and E2F7 mRNA and protein levels were measured in these cells. The data are shown as the means ± SD and were analyzed by one-way ANOVA. *P  <  0.05, **P  <  0.01, ***P  <  0.001, ****P  <  0.0001, ns: no significant difference.

To further explore the specific mechanism by which HuR plays a role in MM, we used NCI-H929 cells transfected with shNC or shHuR lentiviruses for RNA sequencing (RNA-seq). Bubble plots of the results of gene set enrichment analysis (GSEA) of the differentially expressed genes (DEGs) identified via RNA-seq revealed the top ten gene sets, with the “E2F targets” gene set ranked first (Fig. 5a, b). We further analyzed the downregulated genes after HuR knockdown as well as the genes of the E2F family from the RNA-seq results and found that the gene expression of E2F7 was more significantly decreased (Figs. 5c and S5a). In HuR-knockdown NCI-H929 and OPM2 cells, we further validated the mRNA expression levels of HuR and E2F family-related genes via qRT‒PCR; similarly, we found that E2F7 expression was significantly downregulated after HuR knockdown (Fig. 5d). To investigate the correlation between E2F7 and HuR, we used the GSE57317 database for analysis and found that the expression levels of E2F7 and HuR were positively correlated in MM (Fig. 5e). Further investigation revealed that the mRNA and protein levels of E2F7 decreased with HuR knockdown and that the protein level increased with HuR overexpression (Fig. 5d, f, g). We then treated NCI-H929 and OPM2 cells with CMLD-2 at the indicated concentrations and similarly found that the mRNA and protein levels of E2F7 were downregulated (Fig. 5h, i). Taken together, the results show that E2F7 is the main downstream molecule of HuR in MM cells.

### HuR regulates E2F7 by stabilizing its mRNA

**Fig. 6:**
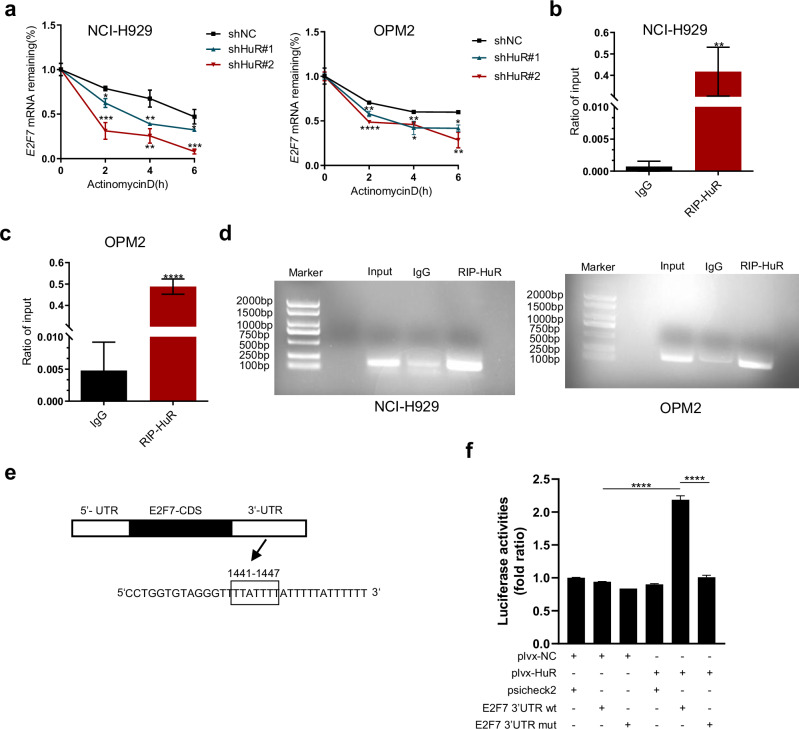
HuR regulates E2F7 by stabilizing its mRNA. **a** HuR-knockdown H929 and OPM2 cells were treated with actinomycin D for 0, 2, 4, and 6 h. E2F7 mRNA levels were measured at corresponding time points via qRT‒PCR. **b**, **c** RIP‒qPCR was performed to detect the interaction between HuR and E2F7 mRNA in NCI-H929 and OPM2 cells. **d** The interaction between HuR protein and E2F7 mRNA in NCI-H929 and OPM2 cells was detected by RIP-PCR and agarose gel electrophoresis. **e** The 3′-UTR sequence of E2F7 mRNA containing the HuR binding site is shown, and this site was predicted by an online website (https://rbpmap.technion.ac.il/index.html). CDS: coding sequence. **f** HEK293T cells were cotransfected with luciferase reporter plasmids (psiCHECK2, E2F7 3′-UTR wt, or E2F7 3′-UTR mut) and plvx-HuR or plvx-NC plasmids. Luciferase activity was measured 48 h after transfection. The data are shown as the means ± SDs, and statistical analysis was performed via Student’s *t*-test (**b**, **c**) or one-way ANOVA (**a**, **f**). *P < 0.05, **P < 0.01, ***P < 0.001, ****P < 0.0001.

HuR, an RNA-binding protein, has been reported to bind to the 3’-UTR of target mRNAs, thereby affecting mRNA stability [24, 32]. Research has revealed that E2F7 is the main downstream molecule of HuR in MM. We next further explored the related regulation of E2F7 by HuR. First, we knocked down HuR in NCI-H929 and OPM2 cells and added Act D to detect the degradation time of E2F7 mRNA via qRT‒PCR. The results revealed that the degradation rate of E2F7 mRNA was significantly greater in the HuR knockdown group than in the shNC group (Fig. 6a). These results suggest that HuR plays an important role in maintaining E2F7 mRNA stability. To determine whether this HuR directly affects the maintenance of E2F7 mRNA stability, we next performed RNA-binding protein immunoprecipitation (RIP) experiments and found that HuR can bind to the 3′-UTR of E2F7 mRNA (Fig. 6b, c). We further performed RIP-PCR experiments and similarly found enrichment of HuR in the 3′-UTR of E2F7 mRNA (Fig. 6d). To further investigate the major sites of HuR binding to the 3′-UTR of E2F7 mRNA, we used online prediction tools to predict the motif of HuR binding to the 3′-UTR of E2F7 mRNA (Fig. S5b). Next, we performed luciferase reporter assays using a dual fluorescence reporter system for the psiCHECK2 plasmid. We found that cotransfection of the pLVX-HuR plasmid resulted in increased luciferase activity in the E2F7 3′-UTR wt construct but not in the E2F7 3′-UTR mut reporter construct (Fig. 6f). These results indicate that HuR directly binds to the 3′-UTR of E2F7 mRNA, causing changes in E2F7 expression levels.

### E2F7 contributes to the effects of HuR in MM as a downstream molecule

**Fig. 7:**
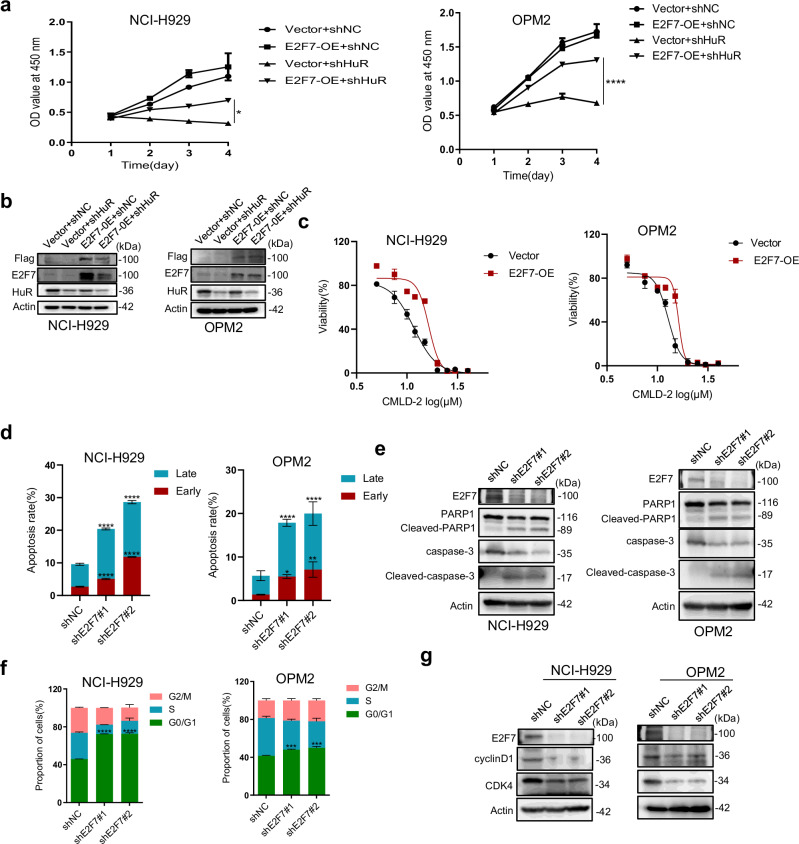
E2F7 contributes to the effects of HuR in MM as a downstream molecule. **a** Proliferation of NCI-H929 and OPM2 cells was examined for 4 consecutive days with CCK-8 assays after co-infection with the following lentiviruses: vector or E2F7-OE (plvx-E2F7-flag-puro) and shNC or shHuR. **b** WB was used to detect protein levels, including those of Flag, HuR, and E2F7, in cells transfected with the above lentiviruses. **c** NCI-H929 and OPM2 cells transfected with vector or E2F7-OE were treated with CMLD-2 at the indicated concentrations for 48 h, and the viability of these cells was tested by CCK-8 assays. **d** After 48 h of lentiviral infection and E2F7 knockdown in NCI-H929 and OPM2 cells, the early and late apoptosis rates were analyzed. **e** Apoptosis-related protein (PARP1, cleaved PARP1, caspase 3, and cleaved caspase 3) levels following E2F7 knockdown in NCI-H929 and OPM2 cells were measured using WB. **f** In NCI-H929 and OPM2 cells, flow cytometry was used to assess the cell cycle after E2F7 knockdown. **g** G_0_/G_1_ phase-associated protein (CDK4 and cyclin D1) levels following E2F7 knockdown in NCI-H929 and OPM2 cells were determined using WB. The data are shown as the means ± SD and were analyzed by one-way ANOVA. *P < 0.05, **P < 0.01, ***P < 0.001, ****P < 0.0001.

The above studies demonstrate that E2F7 may act as a major downstream molecule of HuR. As an RNA-binding protein, HuR has previously been reported to bind mRNA for E2F1, E2F2, and E2F3 of the E2F family in malignant peripheral nerve sheath tumors [8]. Combined with our RNA-seq data, we further explored the functions of E2F1, E2F2, E2F3, and E2F8 in MM. By analyzing data from the MMRF-CoMMpass database, we found that E2F1, E2F2, and E2F8 were associated with a poor prognosis in MM patients (Fig. S6a). We subsequently overexpressed E2F1, E2F2, E2F3, E2F7, and E2F8 in NCI-H929 and OPM2 cells (Fig. S6b). We found that E2F1, E2F2, E2F3, and E2F8 could not reverse the inhibition of MM proliferation caused by the knockdown of HuR (Fig. S6c–g). In addition, under the premise of clarifying the efficiency of E2F7 overexpression and HuR knockdown, we found that the overexpression of E2F7 can partially rescue the proliferation inhibition caused by the knockdown of HuR (Figs. 7a, b and S7a). In addition, we used CCK-8 assays to detect the viability of E2F7-overexpressing NCI-H929 and OPM2 cells at 48 h after CMLD-2 treatment and found that the cell lines overexpressing E2F7 had significantly decreased drug sensitivity to CMLD-2 (Figs. 7c and S7b). We found that the overexpression of E2F7 partially alleviated the apoptosis induced by HuR knockdown and CMLD-2 treatment (Fig. S7c–f). In parallel, simultaneous overexpression of HuR and E2F7 in NCI-H929 and OPM2 cells had a more significant effect on promoting cell proliferation than did overexpression of HuR alone (Fig. S7g, h). These results further demonstrate that E2F7 contributes to the effects of HuR in MM.

### E2F7 knockdown has anti-MM effects in vitro and in vivo

E2F7 acts as a transcription factor of the E2F family, but its specific effects on MM are unknown. To further investigate the role of E2F7 in MM cells, data from the MMRF-CoMMpass dataset were analyzed and showed that higher E2F7 mRNA levels were associated with poorer overall survival (Fig. S7i). In addition, CD138-positive cells from GSE46816 or BMMCs from MM patients presented higher E2F7 expression levels than did CD138-negative cells (Fig. S7j, S8b). For further exploration, we performed loss-of-function experiments in NCI-H929 and OPM2 cells with relatively high E2F7 expression levels (Fig. S8a). We found that E2F7 knockdown suppressed proliferation (Fig. S8c–e), facilitated apoptosis (Figs. 7d and S8f) and induced G_0_/G_1_ phase arrest (Figs. 7f and S8g) in MM cells, accompanied by increases in the levels of the apoptosis-related proteins cleaved PARP1 and caspase 3 and decreases in the levels of the G0/G1 phase-related proteins CDK4 and cyclin D1 (Fig. 7e, g). Then, we subcutaneously inoculated the NCI-H929 and OPM2 cell lines transfected with lentiviruses (shNC, shE2F7#1, or shE2F7#2) into the flanks of NOG mice to construct xenograft mouse models. At the end of the experiment, we dissected subcutaneous tumors from the mice and then photographed, measured, and weighed the tumors. The results revealed that the tumor volume and weight in the E2F7-knockdown group were significantly lower than those in the shNC group (Fig. S9a–c). We also found a significant decrease in the proportion of Ki67-positive cells and a significant increase in the proportion of cleaved caspase 3-positive cells in tumors from the shE2F7 group compared with those from the shNC group (Fig. S9d). These results illustrate that E2F7 knockdown has significant anti-MM effects, further confirming that E2F7 contributes to the effects of HuR in MM as a downstream molecule.

### Targeting HuR enhances bortezomib-mediated anti-MM effects in vitro and in vivo

**Fig. 8:**
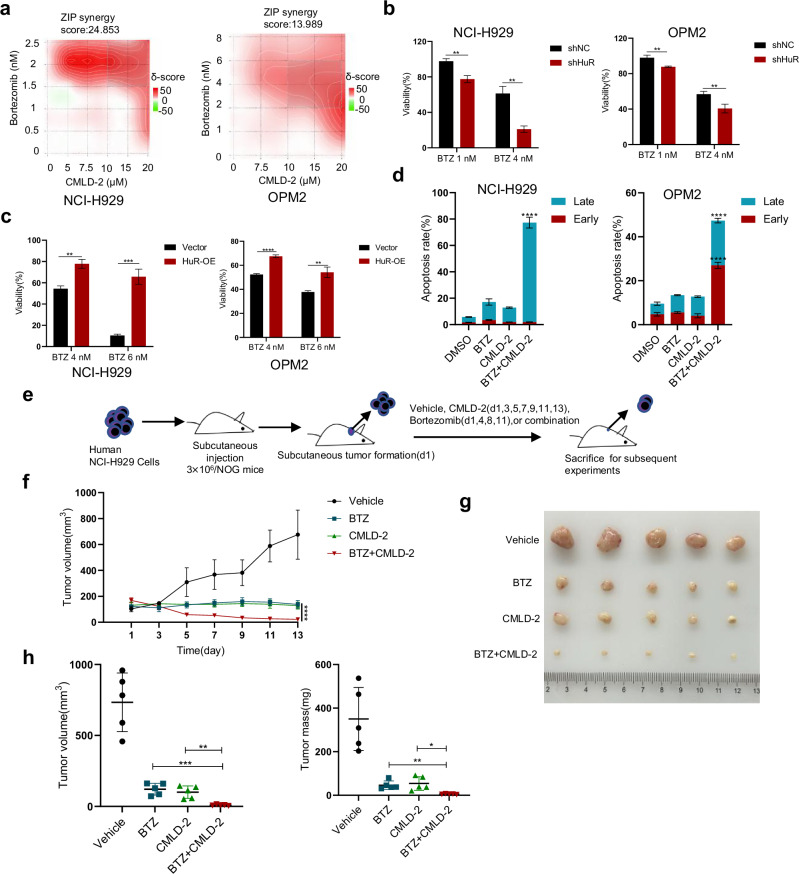
Targeting HuR enhances bortezomib-mediated anti-MM effects in vitro and in vivo. **a** Treatment of NCI-H929 and OPM2 cells with combinations of CMLD-2 and BTZ for 48 h at the indicated concentrations had synergistic effects on cell proliferation. **b**, **c** After HuR-knockdown NCI-H929 and OPM2 cells were treated with bortezomib (BTZ, 1 nM or 4 nM) for 48 h, cell viability was measured with CCK-8 assays. Following the treatment of HuR-overexpressing NCI-H929 and OPM2 cells with BTZ (4 nM or 6 nM) for 48 h, cell viability was determined with CCK-8 assays. **d** NCI-H929 and OPM2 cell lines were treated with BTZ (3 nM), CMLD-2 (15 μM), or a combination for 24 h. The early and late apoptosis rates were analyzed by Annexin V/PI double staining. **e**, **f** NCI-H929 cells were inoculated subcutaneously into the flanks of NOG mice (3 × 10^6^ cells per mouse). After the subcutaneous tumors were measured (d1), the above mice were randomly divided into four groups (*n* = 5 per group). Tumor volume was measured and recorded for NCI-H929 xenograft mice treated with vehicle, CMLD-2 (20 mg/kg, once every other day, intraperitoneally), BTZ (1 mg/kg, d 1, d 4, d 8, and d 11, intravenously), or CMLD-2 + BTZ. **g** These subcutaneous tumors were dissected from mice in each group and photographed at the end of the experiment (d 14). **h** Subcutaneous tumors from the above groups of mice were measured, weighed, and compared. The data are shown as the means ± SD and were analyzed by Student’s *t*-test (**b**, **c**) or one-way ANOVA (**d**, **f**, **h**). *P < 0.05, **P < 0.01, ***P < 0.001, ****P < 0.0001.

Bortezomib (BTZ), dexamethasone, and lenalidomide constitute the backbone of MM therapy [1]. To further explore the therapeutic value of CMLD-2 for MM, we conducted CCK-8 assays to test the combined effects of CMLD-2 and bortezomib, dexamethasone, or lenalidomide. We found that the combination of CMLD-2 and dexamethasone or lenalidomide had additive effects (Fig. S10a, b). The results revealed that CMLD-2 exhibited significant synergistic effects with BTZ in NCI-H929 and OPM2 cells (Fig. 8a). As shown in Fig. 1c, the expression level of HuR was associated with the therapeutic efficacy of bortezomib. Further exploration revealed that sensitivity to bortezomib was significantly decreased by HuR overexpression in NCI-H929 and OPM2 cells and that sensitivity to bortezomib was increased by HuR knockdown (Fig. 8b, c). Moreover, compared with CMLD-2 or BTZ alone, the combination of CMLD-2 and BTZ significantly increased the apoptosis rate of NCI-H929 and OPM2 cells (Figs. 8d and S10c). Interestingly, we found that the E2F7 protein level was significantly lower in the combination group than in the single-agent group (Fig. S10d). These results indicate that E2F7 plays an important role in the synergistic anti-MM effects of CMLD-2 and BTZ.

To further validate the in vivo effects of the CMLD-2 and BTZ drug combination, we constructed a subcutaneous tumor formation model in NOG mice with NCI-H929 cells and randomly divided them for administration, as shown in Fig. 8d. Encouragingly, we confirmed the potent antitumor activity of CMLD-2 in vivo. Moreover, compared with CMLD-2 or BTZ alone, the combination of CMLD-2 and BTZ significantly reduced tumor growth (Fig. 8e–h). In addition, no statistically significant weight loss at the end of the experiment was observed in any mouse after treatment (Fig. S10e). Subsequent IHC staining revealed that compared with CMLD-2 or BTZ alone, the combination of CMLD-2 and BTZ significantly decreased the Ki67 level and increased the cleaved caspase 3 level (Fig. S10f). Taken together, these results show that targeting HuR enhances bortezomib-mediated anti-MM effects in vitro and in vivo.

## Discussion

Many RBPs play important roles in tumor development and tumor cell survival in the tumor microenvironment [7]. Many RBPs, such as heterogeneous nuclear ribonucleoprotein U (hnRNPU) [33], RNA-binding motif protein 39 (RBM39) [34], and cytoplasmic polyadenylation element-binding protein 2 (CPEB2) [35], are associated with MM. HuR has been extensively studied as an RBP in a variety of tumor contexts [36]. However, its role in MM is unknown. In the present study, we found that HuR was upregulated in MM patients and that higher levels of HuR in MM patients were correlated with a poorer prognosis. Patient-derived CD138-positive BMMCs presented higher HuR expression than did CD138-negative BMMCs. HuR knockdown or treatment with CMLD-2, an inhibitor of HuR, inhibited cell proliferation, facilitated apoptosis, and caused G_0_/G_1_ cell cycle arrest in MM cells. The overexpression of HuR promotes MM cell proliferation in vitro and in vivo. Previous studies have reported that HuR leads to treatment resistance by affecting the stability of target genes in colorectal cancer, breast cancer, and prostate cancer [17, 37, 38]. By analyzing GSE9872 data, we found that MM patients who responded better to bortezomib had lower HuR levels. Furthermore, we demonstrated that the sensitivity of MM cells to bortezomib increased with HuR knockdown but decreased with HuR overexpression. The combination of CMLD-2 and bortezomib had synergistic anti-MM effects in vitro and in vivo. The expression level of HuR can be used as a clinical biomarker for the efficacy of bortezomib in MM.

Previous studies have reported that HuR acts in tumors mainly by stabilizing the stability of target mRNAs. For example, in gliomas, HuR mainly affects VEGF-A mRNA stability, thereby affecting angiogenesis [39]. In hepatocellular carcinoma, HuR influences cancer progression by stabilizing BIRC3 mRNA [40]. Further exploring the main mechanism by which HuR plays a role in MM via RNA sequencing showed that E2F7 is the main downstream molecule of HuR. Mechanistically, HuR could increase E2F7 mRNA stability by binding to E2F7 mRNA.

E2F7, a member of the atypical E2F family, plays an important role in DNA damage repair, transcription, and angiogenesis [41]. E2F7 is highly expressed in a variety of tumors, such as colorectal cancer, hepatocellular carcinoma, glioblastoma, and gallbladder cancer, and is associated with a poor prognosis [26, 28, 42, 43]. Similarly, we found that E2F7, a downstream molecule of HuR, plays a critical role in MM. A relatively high E2F7 level was associated with unfavorable outcomes in MM patients. E2F7 knockdown had anti-MM effects both in vitro and in vivo. The overexpression of E2F7 could partially rescue the cell proliferation inhibition and apoptosis caused by HuR knockdown or CMLD-2. These results collectively suggest that the HuR/E2F7 axis plays a critical role in the malignant proliferation of MM cells.

Our study demonstrates that HuR could bind to the E2F7 3′-UTR and positively regulate E2F7 expression by increasing its mRNA stability. Nevertheless, more downstream mechanisms involving E2F7 require further investigation. In glioblastoma, E2F7 acts as a transcription factor and promotes the transcription of EZH2, which inhibits PTEN expression, which in turn activates the AKT/mTOR signaling pathway and ultimately promotes glioblastoma [43]. In view of the above studies, we propose that E2F7 is closely related to PTEN, as well as the AKT/mTOR pathway, in MM cells. The PI3K/AKT/mTOR pathway plays an important role in the pathogenesis of MM. Previous research results from our group have shown that MK2206 can play an anti-MM role in combination with bufalin by inhibiting the AKT/mTOR pathway [44]; additionally, SPAG5 promotes MM by regulating the AKT pathway in MM [45], further indicating the important role of the PI3K/AKT/mTOR pathway in MM. Thus, the biological function of E2F7 in MM, in relation to the PI3K/AKT/mTOR pathway, requires further investigation.

Interestingly, we found that simultaneous overexpression of HuR and E2F7 in MM cells had a more pronounced effect on promoting cell proliferation than did overexpression of HuR alone. However, the overexpression of E2F7 alone did not significantly promote cell proliferation. HuR is highly expressed in MM and shows a clear correlation with E2F7, suggesting that E2F7 may act as a transcription factor for HuR, which leads to high expression of HuR. We conducted further experimental exploration. However, the overexpression and knockdown of E2F7 in NCI-H929 and OPM2 cells did not affect the protein levels of HuR (data not shown). Therefore, we speculated that there may be other molecules that affect their interaction, and the above phenomenon deserves further exploration. We will explore the mechanisms involved in the upstream regulation of HuR expression levels in MM and downstream protein degradation in our next study.

## Conclusions

In conclusion, the present study revealed that the HuR/E2F7 axis is involved in MM proliferation. Mechanistically, HuR binds to the E2F7 3′-UTR and upregulates E2F7 expression by stabilizing its mRNA. Both CMLD-2 treatment and HuR knockdown significantly synergized with the anti-MM effect of bortezomib. Therefore, targeting the HuR/E2F7 axis, particularly in combination with bortezomib, has promise as a strategy for treating MM.

## Supplementary information

Supplementary figure

Supplementary Information

## Data Availability

The data are available from the corresponding author upon reasonable request.
