# Supplementary material for: Targeting the HuR/E2F7 axis synergizes with bortezomib against multiple myeloma
Source: Acta Pharmacol Sin. 2025 Mar 25;46(8):2296–309. doi: 10.1038/s41401-025-01529-3 (PMC12274554; doi:10.1038/s41401-025-01529-3)

Supplementary Figures

Supplementary Figure.1

a

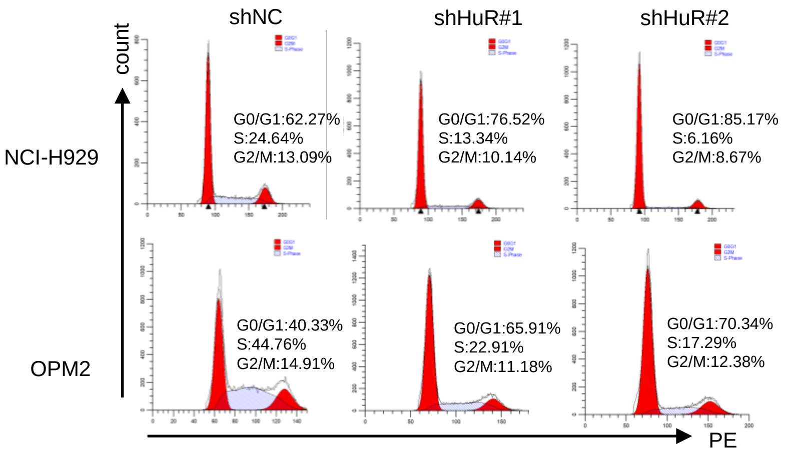

Supplementary Figure.2

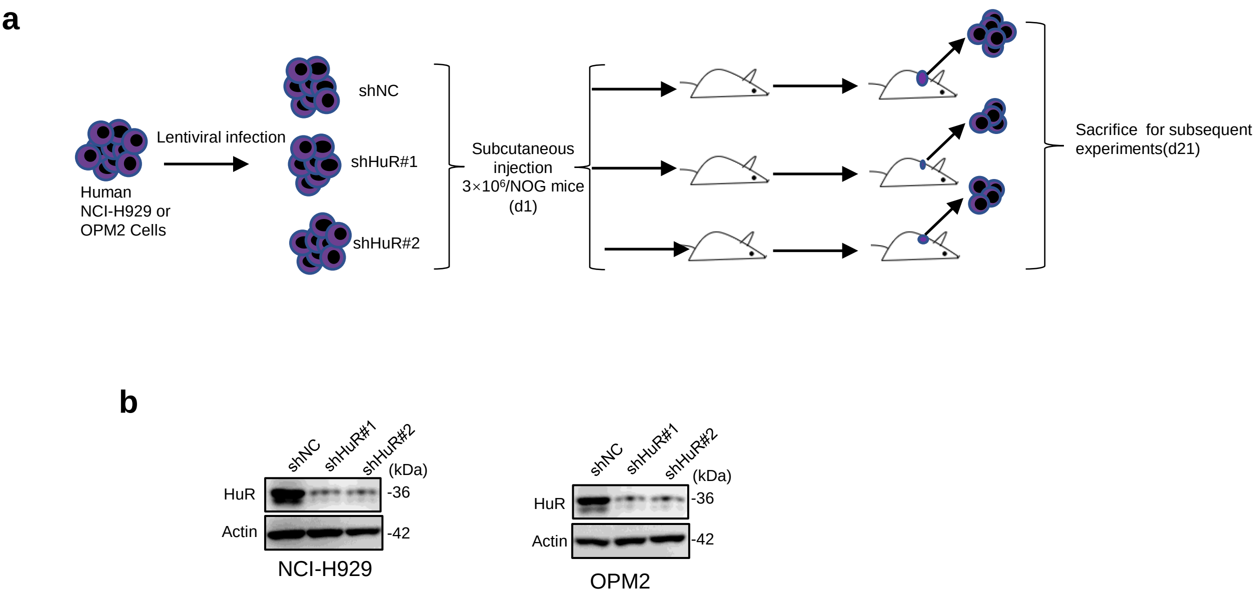

Supplementary Figure.3

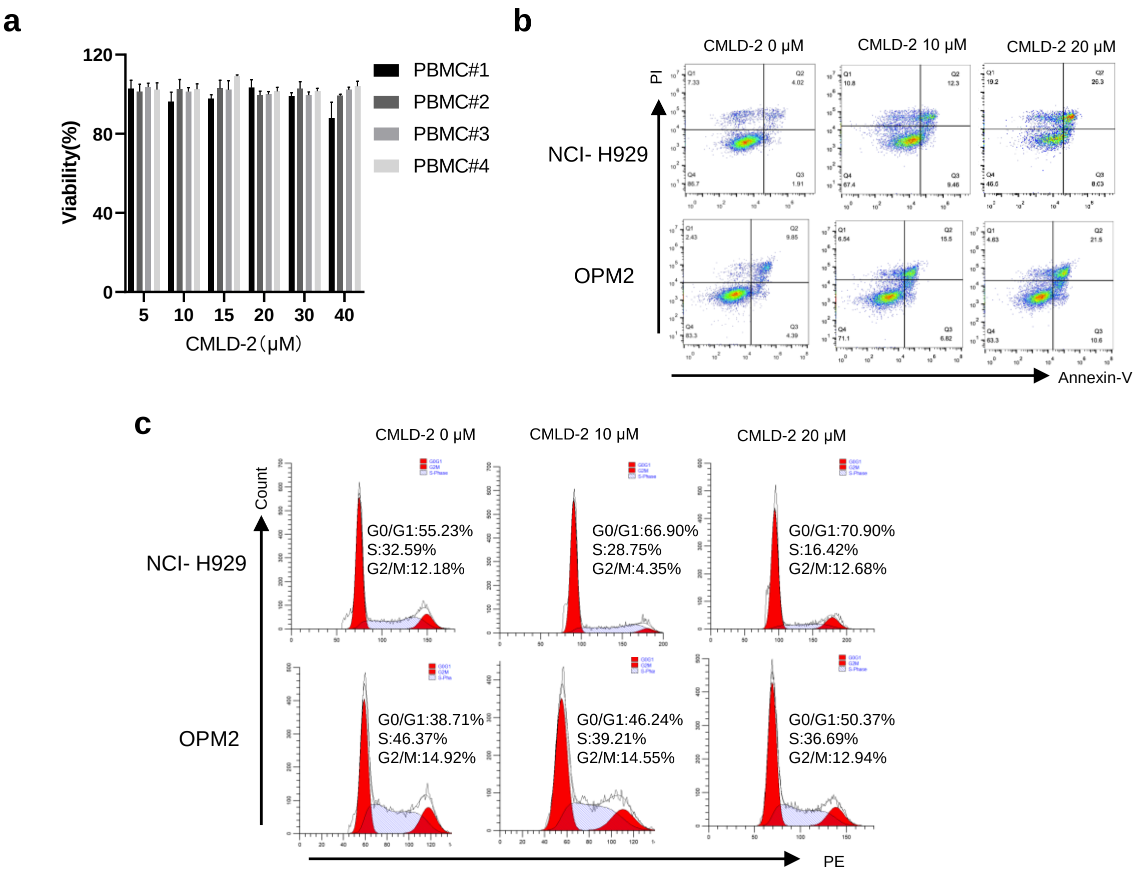

Supplementary Figure.4

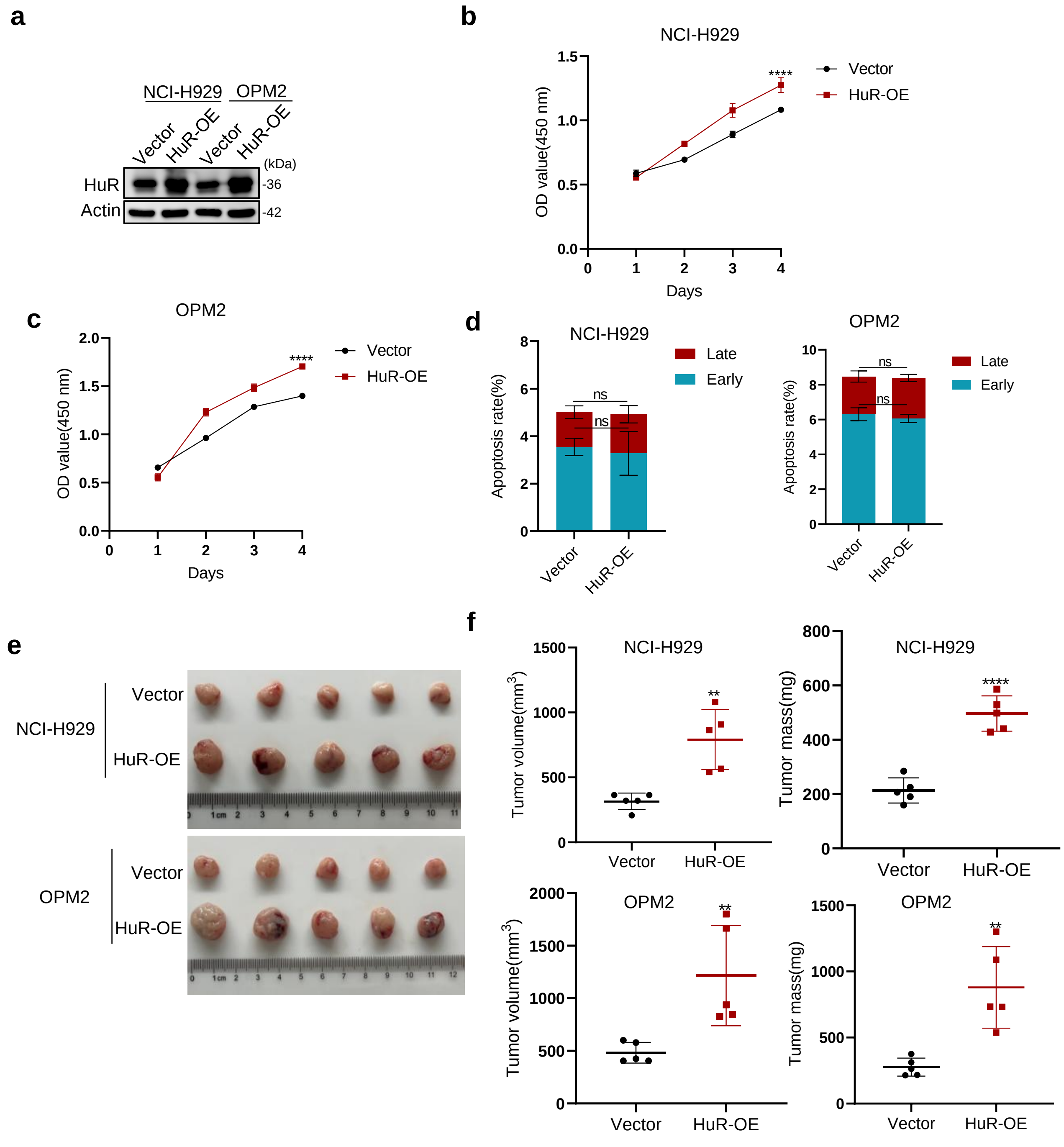

Supplementary Figure.5

a

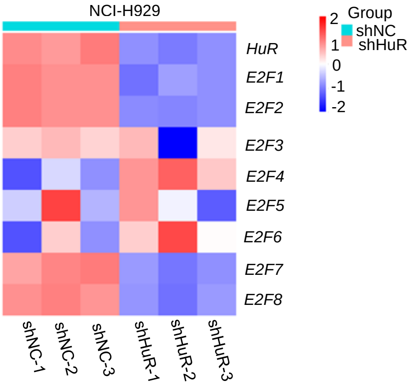

b

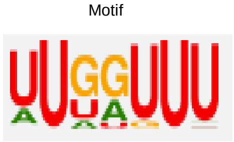

Supplementary Figure.6

a

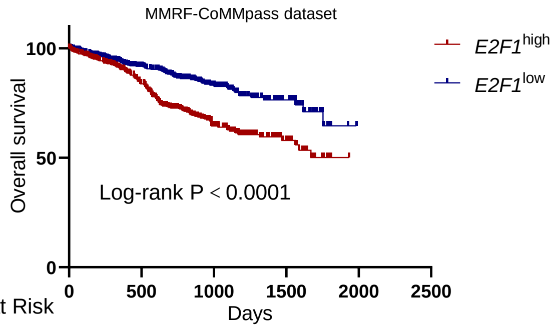

No.at Risk

*E2F1*high	429	290	128	35	1
*E2F1*Low	430	327	164	40	1

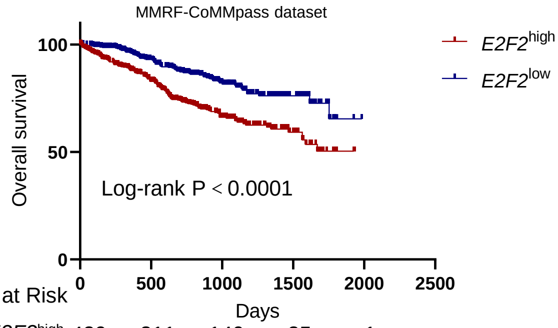

No.at Risk

*E2F2*high	429	311	140	35	1
*E2F2*Low	430	306	154	40	1

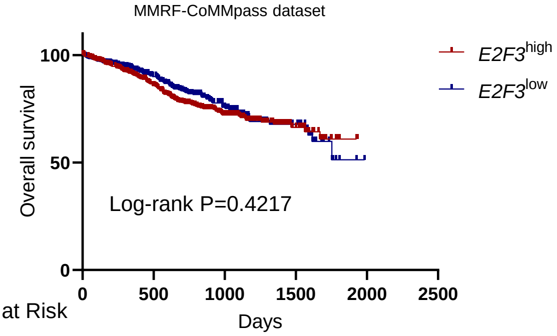

No.at Risk

*E2F3*high	431	331	166	46	1
*E2F3*Low	428	286	126	29	1

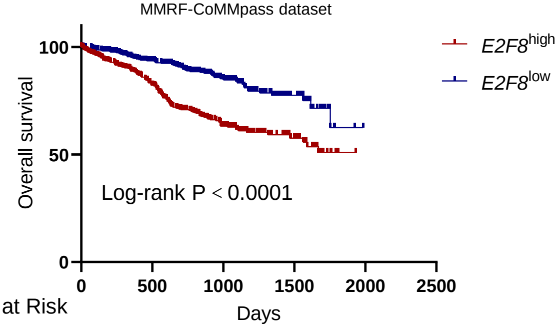

No.at Risk

*E2F8*high	429	303	140	37	1
*E2F8*Low	430	314	152	38	1

Supplementary Figure.6

b

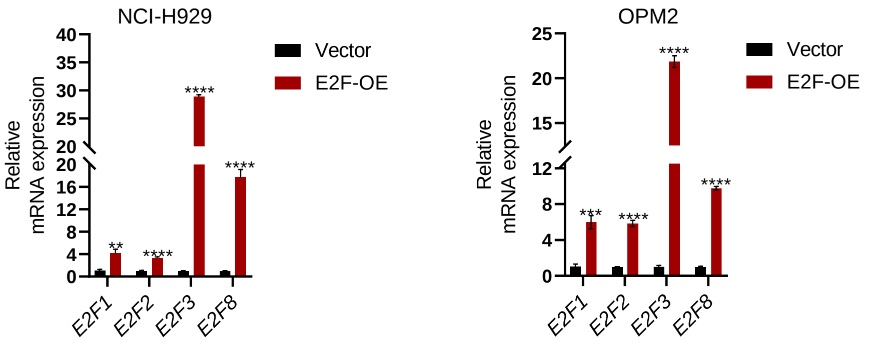

c

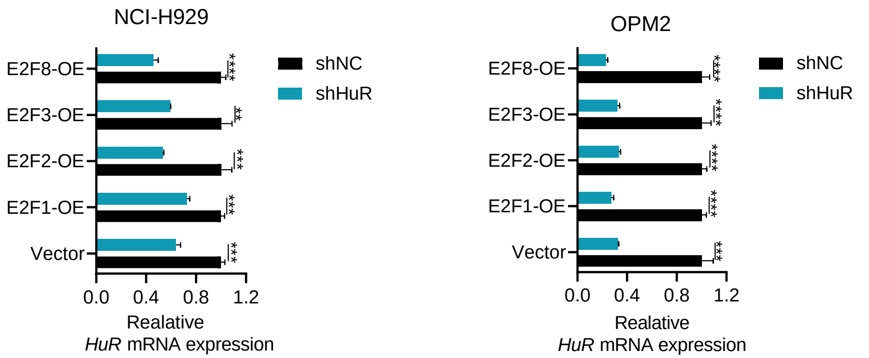

Supplementary Figure.6

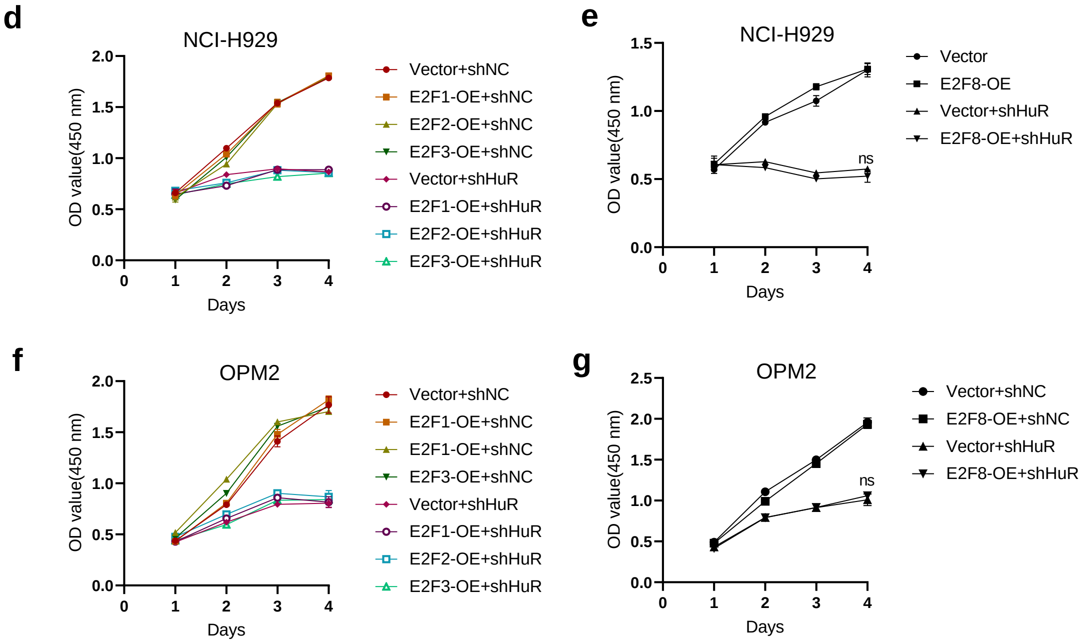

Supplementary Figure.7

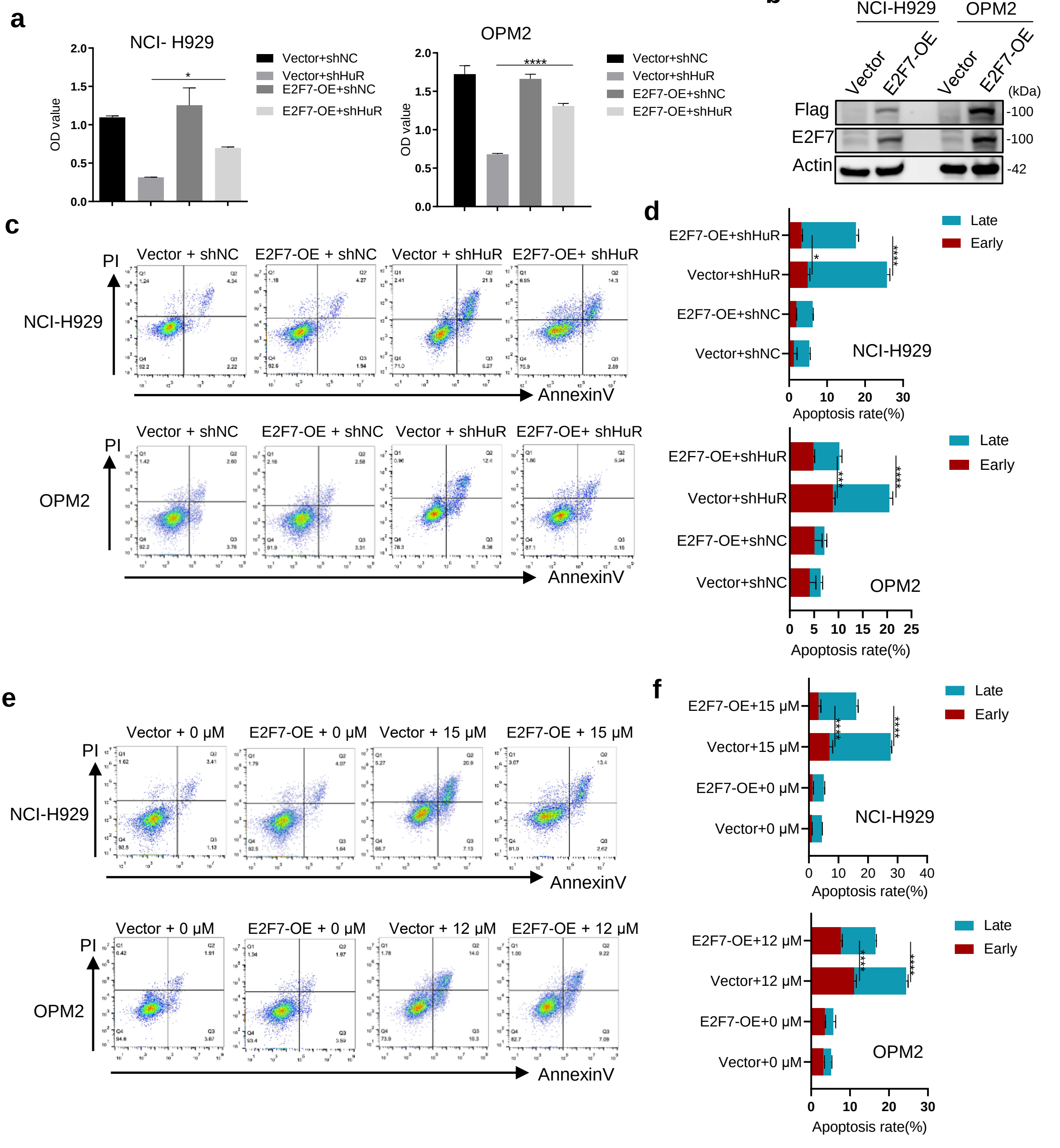

Supplementary Figure.7

g

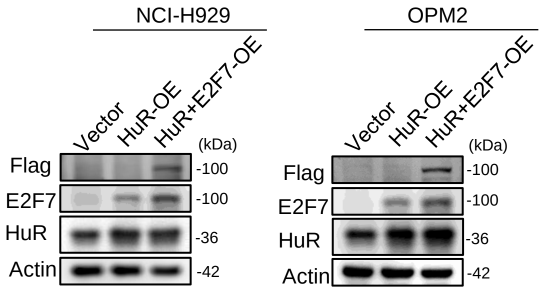

h

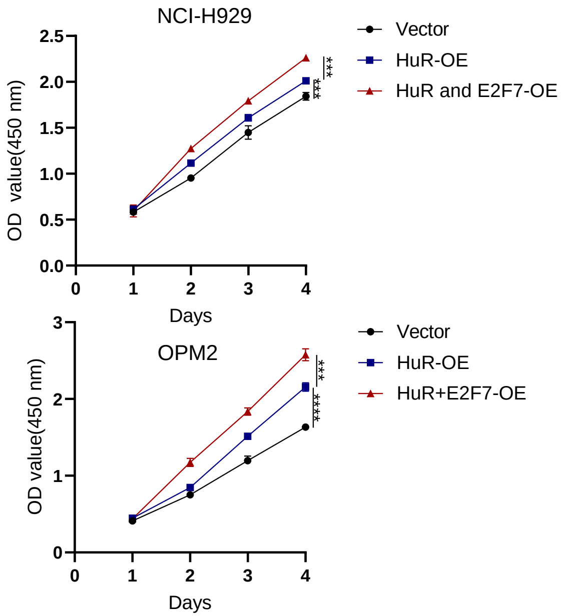

i

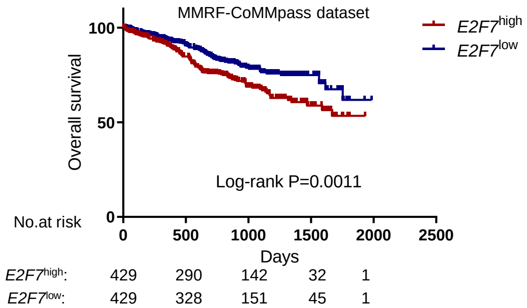

j

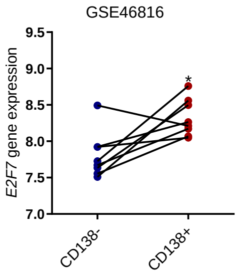

Supplementary Figure.8

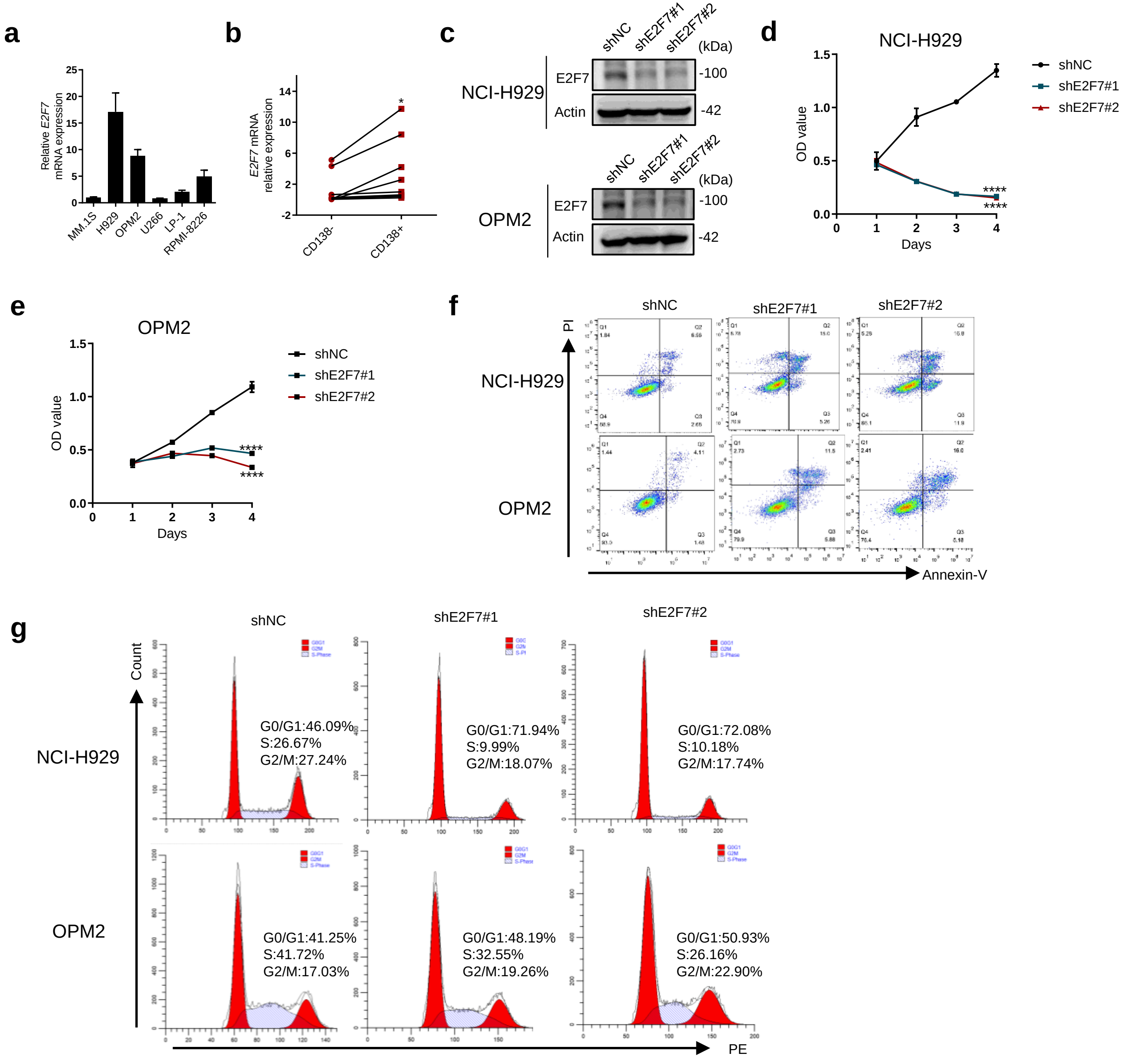

Supplementary Figure.9

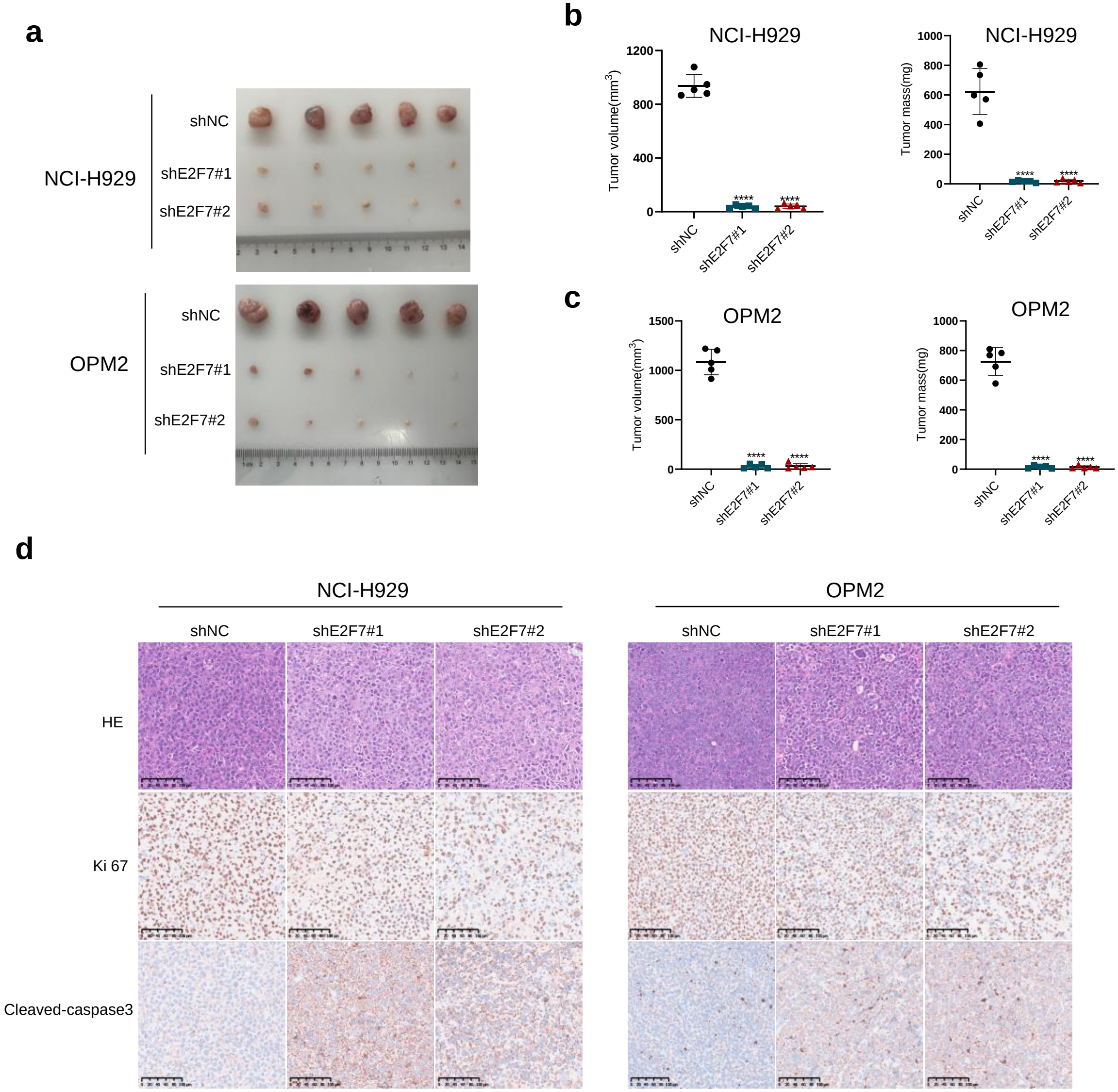

Supplementary Figure.10

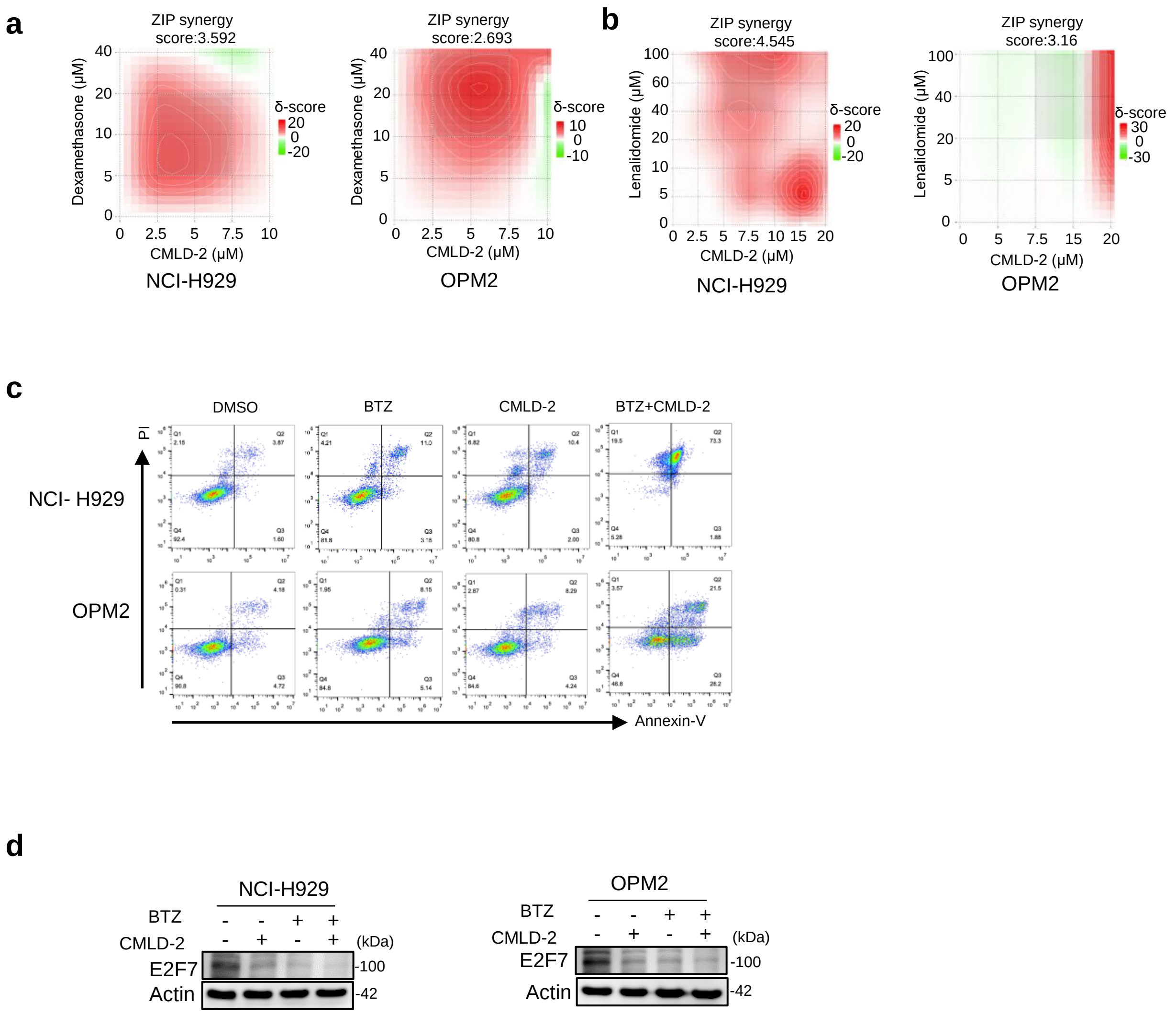

Supplementary Figure.10

e

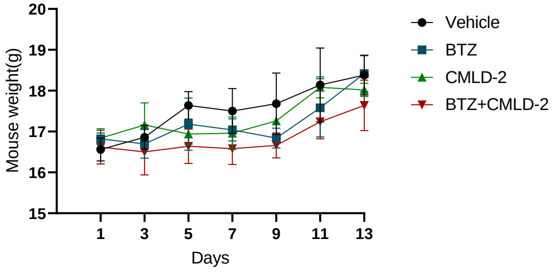

f

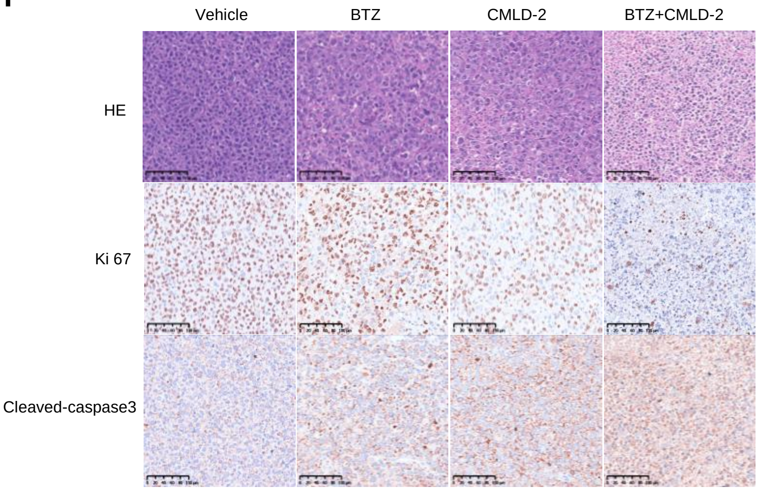

Supplement: Supplementary file 1 — Supplementary figure [file 41401_2025_1529_MOESM1_ESM.pdf]
