# Supplementary material for: Targeting the HuR/E2F7 axis synergizes with bortezomib against multiple myeloma
Source: Acta Pharmacol Sin. 2025 Mar 25;46(8):2296–309. doi: 10.1038/s41401-025-01529-3 (PMC12274554; doi:10.1038/s41401-025-01529-3)
Supplement: Supplementary file 2 — Supplementary Information [file 41401_2025_1529_MOESM2_ESM.docx]

**Targeting the HuR/E2F7 axis synergizes with bortezomib against multiple myeloma**

| Supplementary Table 1 |  |
| --- | --- |
|  |  |
| human qRT-PCR Primers | sequence |
| E2F1-F | ACGCTATGAGACCTCACTGAA |
| E2F1-R | TCCTGGGTCAACCCCTCAAG |
| E2F2-F | CGTCCCTGAGTTCCCAACC |
| E2F2-R | GCGAAGTGTCATACCGAGTCTT |
| E2F3-F | GTATGATACGTCTCTTGGTCTGC |
| E2F3-R | CAAATCCAATACCCCATCGGG |
| E2F4-F | GCAGACCCCACAGGTGTTTT |
| E2F4-R | GCTCCGAGCTCATGCACTCT |
| E2F5-F | TTGCTTTAATGGTGATACACTTTTGG |
| E2F5-R | TCTGACCCATTTCTGGAATGG |
| E2F6-F | GAAAATGAAAGACTAGCATATGTGACC |
| E2F6-R | CTTTAACTGCAATGACGATCTGTTC |
| E2F7-F | ATCAAGGATGGCCCCGAAGA |
| E2F7-R | GTTTTCTGCTTGGCCTTTGC |
| E2F8-F | AAAATGAAAAATCTGGAGTTCCTCC |
| E2F8-R | CTGATCTGCGAACAGGATATTAAAAC |
| HuR-F | GGGTGACATCGGGAGAACG |
| HuR-R | CTGAACAGGCTTCGTAACTCAT |
| E2F7-F1 | AAAGGGACTATTCCGACCCAT |
| E2F7-R1 | ACTTGGATAGCGAGCTAGAAACT |
| β-actin-F | CATGTACGTTGCTATCCAGGC |
| β-actin-R | CTCCTTAATGTCACGCACGAT |
|  |  |

**Supplementary Figure legends**

**Supplementary Figure.1. HuR knockdown causes G0/G1 phase arrest in MM cells**

**a** The cell cycle of NCI-H929 and OPM2 cells with HuR knockdown was detected by flow cytometry, and the proportion of cells in G0/G1, S, and G2 phase were analyzed using ModiFit software.

**Supplementary Figure.2. The construction of NCI-H929 or OPM2 based xenograft NOG mice a** NCI-H929 or OPM2 cells infected with shNC, shHuR#1, and shHuR#2 lentiviruses were subcutaneously inoculated into the flank (d1) of NOG mice (3 × 10^6^ cells/mouse). At the end of the experiment (d21), these mice were sacrificed for subsequent experiments. **b** Expression levels of HuR in proteins from subcutaneous tumors of mice in each of the above groups was measured by WB.

**Supplementary Figure.3. CMLD-2 facilitates apoptosis and causes G0/G1 phase arrest in MM cells without affecting healthy PBMCs.**

**a** PBMCs isolated from four healthy donors (PBMC#1, #2, #3 and #4) were treated with CMLD-2 at the indicated concentrations for 48 h, and cell viability was then assessed via CCK-8 assays. **b** Apoptosis rates of NCI-H929 and OPM2 treated with CMLD-2 (0 μM, 10 μM, 20 μM) for 48 h were detected by Annexin V/PI double staining. **c** The cell cycle of NCI-H929 and OPM2 cells treated with CMLD-2 (0 μM, 10 μM, 20 μM) for 48 h was detected by flow cytometry, and the proportion of cells in G0/G1, S, and G2 phase were analyzed using ModiFit software.

**Supplementary Figure.4. Overexpression of HuR promotes MM cell proliferation in vitro and in vivo.**

**a** Protein levels of HuR were detected by WB after lentivirus (Vector, HuR-OE) infection for 48h in NCI-H929 and OPM2 cells. **b-c** CCK-8 assays were used to detect cell proliferation in NCI-H929 and OPM2 cells after overexpression of HuR for 4 consecutive days. **d** After 48 h of lentiviral infection with overexpression of HuR, the early apoptotic cells (Annexin V positive and PI negative) and late apoptotic cells (Annexin V positive and PI positive) of NCI-H929 and OPM2 cells were used for apoptosis rate analysis. **e** NCI-H929 and OPM2 cells infected with lentiviral (Vector or HuR-OE) were inoculated subcutaneously into NOG mice to construct xenograft mouse models (3 × 10^6^ cells/mouse, n=5). These subcutaneous tumors were dissected from each group of mice and photographed at the end of the experiment(d14). **f** Subcutaneous tumors from the above groups of mice were measured, weighed and compared. The data are shown as the means ± SD, statistical analysis was performed via Student's t test. ***P* < 0.01, *****P*< 0.0001.

**Supplementary Figure.5. The heatmap of RNA-sequencing and the motif predicted online.**

**a** Heatmap of RNA sequencing E2F family expression data in NCI-H929 infected with shNC, shHuR lentiviruses (n = 3). **b** The motif about HuR binding with E2F7 mRNA from online website (https://rbpmap.technion.ac.il/index.html).

**Supplementary Figure.6. E2F1, E2F2, E2F3 and E2F8 cannot rescue the inhibition of proliferation caused by knockdown of HuR in MM cells.**

**a** In the MMRF-CoMMpass database, the log-rank test was used to statistically analyze the overall survival in MM patients with high expression and low expression of E2F1, E2F2, E2F3 and E2F8. **b** After NCI-H929 and OPM2 cells were infected with the following lentiviruses, including Vector or E2F1-OE, E2F2-OE, E2F3-OE, E2F8-OE for 48 h, E2F1, E2F2, E2F3 or E2F8 mRNA levels in the above cells were detected by qRT-PCR, respectively. **c** After NCI-H929 andOPM2 cells were co-infected with the following lentiviruses, including Vector or E2F1-OE, E2F2-OE, E2F3-OE, E2F8-OE, shNC or shHuR for 48 h, the HuR mRNA levels in the above cells were detected by qRT-PCR, respectively. **d-g** Proliferation of NCI-H929 and OPM2 cells was examined for 4 consecutive days with CCK-8 assays after co-infection with the following lentiviruses, including Vector or E2F1-OE, E2F2-OE, E2F3-OE, E2F8-OE, shNC or shHuR. The data are shown as the means ± SD, statistical analysis between two groups was performed via Student's t test. ***P* < 0.01, ****P*< 0.001, *****P* < 0.0001.

**Supplementary Figure.7.** **E2F7 contributes to the effects of HuR in MM as a downstream molecule and associates with poor prognosis in MM.**

**a** The comparison of NCI-H929 and OPM2 cells proliferation measured by CCK-8 assays on day 4 after co-infection with the following lentiviruses, including Vector, E2F7-OE (plvx-E2F7-flag-puro), shNC or shHuR. **b** The overexpression of E2F7 was confirmed by WB after infection with Vector or E2F7-OE for 48 h in NCI-H929 and OPM2 cells. **c-d** After NCI-H929 and OPM2 cells were co-infected with the following lentiviruses, including Vector, E2F7-OE, shNC or shHuR for 48 hours, the apoptosis of the above cells was detected by flow cytometry using Annexin V/PI double staining. Then the early and late apoptotic cells of the above cells were used for apoptosis rate analysis. **e-f** The E2F7-overexpression NCI-H929 and OPM2 cells were treated with CMLD-2 (0 μM, 15 μM or 12 μM) for 48 h, the apoptosis of the above cells was measured by flow cytometry using Annexin V/PI double staining. Then the early and late apoptotic cells of the above cells were used for apoptosis rate analysis. **g** After NCI-H929 and OPM2 cells were infected with the following lentiviruses, including Vector, HuR-OE or HuR-OE and E2F7-OE for 48 hours, the protein levels of E2F7 and HuR were measured by WB. **h** Proliferation of NCI-H929 and OPM2 cells was examined for 4 consecutive days with CCK-8 assays after infection with the following lentiviruses, including Vector, E HuR-OE or HuR-OE and E2F7-OE. **i** In the MMRF-CoMMpass database, the log-rank test was used to statistically analyze the overall survival in MM patients with high E2F7 expression (E2F7^high^) and lowE2F7 expression (E2F7^low^). **j** The eight paired CD138 + and CD138- cells were analyzed for E2F7 expression in GSE46816 database. The data are shown as the means ± SD, statistical analysis was performed via Student's t test (**j**) or one-way ANOVA (**a**, **d**, **f,** **h**). **P* < 0.05, ****P* < 0.001, *****P* < 0.0001.

**Supplementary Figure.8. E2F7** **knockdown suppresses the proliferation, facilitates apoptosis and induces G0/G1 phase arrest in MM cells.**

**a** Relative expression of E2F7 detected by qRT-PCR in six MM cell lines. **b** The mRNA expression levels of E2F7 were detected by qRT-PCR in CD138 + and CD138− cell populations obtained from BMMCs of MM patients. **c** Knockdown efficiency of E2F7 in NCI-929 and OPM2 was detected by WB. **d-e** CCK-8 assays were used to detect cell proliferation in NCI-H929 and OPM2 cells after knockdown of E2F7 for 4 consecutive days, and Student 's t-test were used to compare proliferation between the two groups on day 4. **f** After 48 h of lentiviral infection with knockdown of E2F7, the apoptosis rate of NCI-H929 and OPM2 cells was detected by flow cytometry using Annexin V/PI double staining. **g** After 48 h of lentiviral infection with knockdown of E2F7, the cell cycle of NCI-H929 and OPM2 cells was detected by flow cytometry. The proportion of cells in G0/G1, S, and G2 phase were analyzed using ModiFit software. The data are shown as the means ± SD, statistical analysis was performed via Student's t test (**b**) or one-way ANOVA (**d**, **e**). **P* < 0.05, *****P* < 0.0001.

**Supplementary Figure.9. E2F7 knockdown exhibits anti-MM effects in vivo.**

(a) NCI-H929 and OPM2 cells infected with lentiviral (shNC, shE2F7#1 and shE2F7#2) were inoculated subcutaneously into NOG mice to construct xenograft mouse models. These subcutaneous tumors were dissected from each group of mice and photographed at the end of the experiment. n=5. (b, c) Subcutaneous tumors from the above groups of mice were measured, weighed and compared. (d) Subcutaneous tumors from three groups of mice, shNC, shE2F7#1, and shE2F7#2, were paraffin-embedded and stained with hematoxylin and eosin (HE), Ki67, and cleaved caspase-3 antibodies (scale 100 μm). The data are shown as the means ± SD, statistical analysis was performed via one-way ANOVA. *****P* < 0.0001.

**Supplementary Figure.10.** **Targeting HuR enhances bortezomib-mediated anti-MM effects in vitro and in vivo.**

**a** Treatment of NCI-H929 and OPM2 cells with combinations of CMLD-2 and dexamethasone at the indicated concentrations for 48 h showed additive effects on cell proliferation. **b** Treatment of NCI-H929 and OPM2 cells with combinations of CMLD-2 and lenalidomide at the indicated concentrations for 48 h showed additive effects on cell proliferation. **c** NCI-H929 and OPM2 cell lines were treated with BTZ (3 nM), CMLD-2 (15 μM), or a combination for 24 h. Apoptosis rate was measured by flow cytometry using Annexin V/PI double staining. **d** After NCI-H929 and OPM2 cells were treated with BTZ (0 nM or 3 nM), CMLD-2 (0 μM or 15 μM), or a combination for 12 h, the protein levels of E2F7 were measured by WB. **e** Body weights of mice were measured and recorded every other day in four groups, including vehicle, BTZ, CMLD-2 and BTZ + CMLD-2. No statistically significant difference in body weight between groups. **f** Subcutaneous tumors from above four groups of mice were paraffin-embedded and stained with hematoxylin and eosin (HE), Ki67, and cleaved caspase-3 antibodies (scale 100 μm). The data are shown as the means ± SD, statistical analysis was performed via one-way ANOVA.
